# Anti-Ro52/TRIM21 seropositivity in interstitial lung disease: effect modification by disease subtype and myositis-specific antibody context

**DOI:** 10.3389/fimmu.2026.1960409

**Published:** 2026-10-16

**Authors:** Junfeng Su, Yan Du, Chen Zhu, Haodong Zhou, Huyan Wang, Yinjun Du, Qiyuan Wang, Zexin Chen, Hanjun Cao, Man Huang, Jing Xue

**Affiliations:** 1 Department of General Intensive Care Unit, the Second Affiliated Hospital of Zhejiang University School of Medicine, Hangzhou, China; 2 Key Laboratory of Multiple Organ Failure (Zhejiang University), Ministry of Education, Hangzhou, Zhejiang, China; 3 Department of Rheumatology, the Second Affiliated Hospital of Zhejiang University School of Medicine, Hangzhou, China; 4 Key Laboratory of Respiratory Disease of Zhejiang Province, Department of Pulmonary and Critical Care Medicine, the Second Affiliated Hospital of Zhejiang University School of Medicine, Hangzhou, China; 5 Department of Pharmacy, the Second Affiliated Hospital of Zhejiang University School of Medicine, Hangzhou, China; 6 Department of Rheumatology, the First Affiliated Hospital of Ningbo University, Ningbo, China; 7 Department of Rheumatology, Ningbo Yinzhou No.2 Hospital, Ningbo, China; 8 Department of Radiology, the Second Affiliated Hospital of Zhejiang University School of Medicine, Hangzhou, China; 9 Department of Clinical Epidemiology & Biostatistics, the Second Affiliated Hospital of Zhejiang University School of Medicine, Hangzhou, China; 10 Department of Critical Care Medicine, The First People’s Hospital of Jingdezhen, Jiangxi, China

**Keywords:** anti-Ro52 antibodies, connective tissue disease, interstitial lung disease, mortality, myositis, TRIM21

## Abstract

**Background:**

Anti-Ro52/TRIM21 shows contradictory prognostic associations in interstitial lung disease (ILD), protective in some cohorts and adverse in others. We evaluated whether it acts as an independent predictor or a phenotypic marker and whether its effect is modified by disease subtype and myositis-specific antibodies.

**Methods:**

We retrospectively identified 538 adults with radiologically confirmed interstitial lung disease (228 anti-Ro52-positive) from a 2010–2020 single-center cohort. Cox proportional-hazards models were sequentially adjusted for demographic, smoking, malignancy, and pulmonary-function covariates. Pre-specified effect modification by connective tissue disease (CTD) status and myositis-specific antibodies was assessed by likelihood-ratio interaction tests with false-discovery-rate correction. Robustness to missing data was supported by multiple imputation (*m* = 20) and a missing-not-at-random (MNAR) tipping-point analysis.

**Results:**

Ro52 positivity was associated with younger age, CTD predominance, and lower complement levels. Pre-specified effect modification was suggested: anti-Ro52 trended toward lower mortality in CTD-ILD (HR 0.73, 95% CI 0.49–1.07, *P* = 0.104) but did not independently increase mortality within the anti-MDA5-positive subgroup (HR 1.73, 95% CI 0.66–4.54). Instead, the adverse prognostic effect of anti-MDA5 was numerically greater among anti-Ro52-positive than Ro52-negative patients (MDA5 HR 10.57, 95% CI 3.60–31.03 vs. 2.56, 95% CI 0.97–6.74), although the formal interaction term did not reach statistical significance (*P* = 0.055). The fully adjusted (Model 6) HR was 0.72 (95% CI 0.52–0.99; *P* = 0.045); further adjustment for CTD status (Model 7) attenuated the association to non-significance (HR 0.75, 95% CI 0.54–1.04, *P* = 0.091), and IPCW weighting and time-varying-age analyses were likewise non-significant; malignancy independently predicted mortality (HR 1.81, 95% CI 1.15–2.88; *P* = 0.012).

**Conclusion:**

Anti-Ro52 is a context-dependent phenotypic marker of immune-mediated ILD, not a universal prognostic biomarker. Its prognostic association is heterogeneous across ILD phenotypes: it is not independently protective in CTD-ILD (adjusted HR 0.73, *P* = 0.104), whereas the adverse prognostic effect of anti-MDA5 was numerically greater among anti-Ro52-positive than Ro52-negative patients (MDA5 HR 10.57 vs. 2.56), although this interaction did not reach statistical significance (*P* = 0.055); CTD-status adjustment similarly attenuated the Ro52 association (HR 0.75, *P* = 0.091). These findings support stratified prognostic models integrating Ro52 with autoantibody and disease-subtype context.

## Introduction

The clinical significance of anti-Ro52 antibodies remains controversial in interstitial lung disease (ILD), a heterogeneous group of disorders whose prognosis varies substantially by etiology (1–3). Idiopathic pulmonary fibrosis (IPF), a progressive fibrosing interstitial pneumonia characterized by the usual interstitial pneumonia (UIP) pattern after exclusion of known causes, carries a median survival of 3–5 years (4), whereas immune-mediated ILD, particularly CTD-ILD, responds to immunosuppression and has superior survival (5). Mechanistically, IPF involves telomere dysfunction and aberrant repair (6), whereas non-UIP patterns (e.g., NSIP) in CTD-ILD respond better to immune modulation (7, 8). These context-dependent differences call for evaluation within a unified cohort.

Part of the Ro ribonucleoprotein particle (9, 10), Ro52 (also known as TRIM21) is the 52-kD component of the SS-A/Ro antigen (11) and interacts with other proteins (12). Anti-Ro52 antibodies have clinical relevance across systemic autoimmune diseases (13) and recognized diagnostic utility (14). Their prognostic implications differ by disease context: in idiopathic inflammatory myopathy (IIM), Ro52 is associated with rapidly progressive ILD and higher mortality (15–18), whereas in systemic sclerosis (SSc)–ILD, it may track disease progression (19–21). These contrasting findings suggest effect modification by disease subtype and autoantibody profile. This concept has not been systematically tested in a unified ILD cohort and is best interpreted within subtype-specific prognostic frameworks (4, 5).

We analyzed 538 ILD patients from a 2010–2020 cohort to (1) define the clinical associations of Ro52, (2) determine its distribution across ILD subtypes and radiological patterns, and (3) evaluate its prognostic significance. We examined whether effect modification by disease subtype and autoantibody profile underlies anti-Ro52’s role: an independent mortality predictor or a context-dependent phenotypic marker of immune-mediated ILD.

## Materials and methods

### Study design and participants

Adult patients with ILD who underwent anti-Ro52 testing at the study center between 2010 and 2020 were identified through the hospital electronic medical record system (EMRS). Eligibility criteria were as follows: (1) a diagnostic code of ILD or interstitial pneumonia among the first three diagnostic code positions, with discharge dates from 1 August 2010 to 1 August 2020; (2) hospitalization ≥2 days; (3) treatment in the rheumatology, pulmonary, or intensive care unit (ICU) departments; (4) anti-Ro52 antibody testing performed during or in temporal proximity to the index hospitalization; and (5) when patients had multiple admissions, the ICU admission record was selected as the index hospitalization to capture the most severe and clinically complete presentation (pulmonary function, autoantibody, and imaging data). The index date was defined as the admission date of this index hospitalization, and anti-Ro52 testing was performed during or in temporal proximity to it. From 3,973 patients retrieved by the interstitial pneumonia/interstitial lung disease diagnostic code at any position, 1,803 were excluded (interstitial pneumonia/ILD not among the first three diagnostic codes), 1,502 were excluded (hospital stay < 2 days), and 130 were excluded (anti-Ro52 not tested; of these, 126 had nonetheless been entered in our data-extraction tables, as detailed in the Limitations), leaving 538 patients for analysis (**Figure 1**). Records were extracted from the EMRS by information-technology staff using structured query language and manually verified by the research team, as our institution has no dedicated research database. The final cohort comprised all eligible patients identified through the EMRS; because the center lacked a dedicated research database, records were assembled in successive extraction waves, including completion of laboratory data for ICU-admitted patients in a later wave, with every wave applying the same objective eligibility criteria independent of Ro52 status or outcome. No *a priori* power calculation was performed.

**Figure 1:**
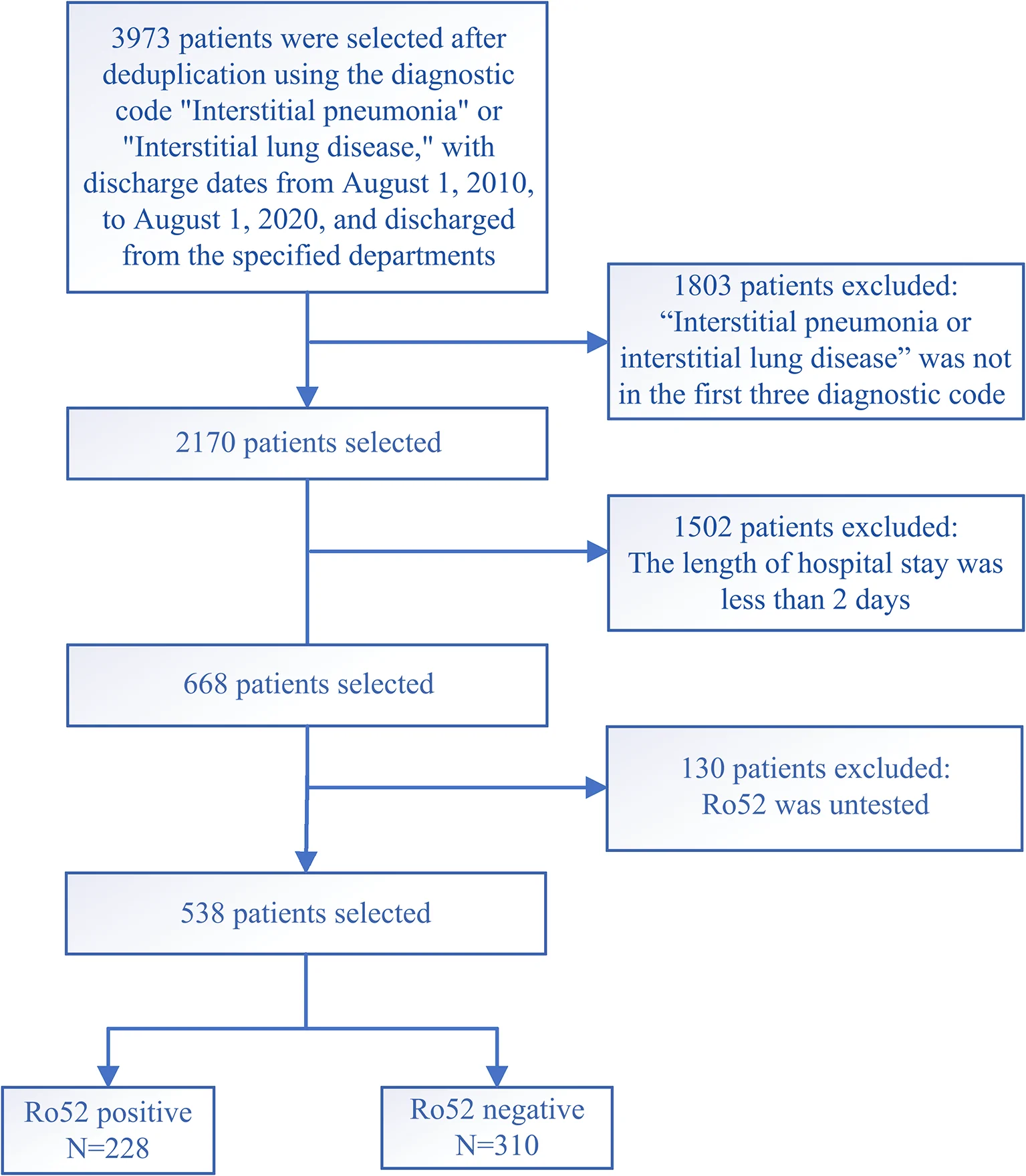
Flow diagram of patient selection. From 3,973 patients (deduplicated to one record per patient) identified in the hospital electronic medical record system by a diagnostic code of interstitial pneumonia or interstitial lung disease at any position, with discharge dates from 1 August 2010 to 1 August 2020, and discharged from the Department of Rheumatology, Department of Pulmonary and Critical Care Medicine, or Department of General Intensive Care Unit, 1,803 patients were excluded because interstitial pneumonia or interstitial lung disease was not among the first three diagnostic code positions, leaving 2,170 patients. After excluding 1,502 patients whose length of hospital stay was less than 2 days, 668 patients remained. After excluding 130 patients for whom anti-Ro52 had not been tested, 538 patients were included, comprising 228 anti-Ro52-positive and 310 anti-Ro52-negative patients. Records were assembled in successive extraction waves applying identical eligibility criteria (see Methods).

### Data collection

Clinical data were extracted from the EMRS by two independent investigators, including demographics, high-resolution computed tomography (HRCT) findings, pulmonary function tests (PFTs), initial ILD diagnosis, and admission laboratory data: autoantibody profiles [anti-Ro52, anti-Ro60, and antinuclear antibody (ANA)], enzymes [creatine kinase (CK), alanine aminotransferase (ALT), lactate dehydrogenase (LDH), and aspartate aminotransferase (AST)], inflammatory markers [C-reactive protein (CRP), ferritin], immunoglobulins (IgA, IgG, IgM), complement (C3, C4), and lymphocyte subsets (T-cell counts, CD4/CD8 ratio), assayed by enzyme-linked immunosorbent assay (ELISA), chemiluminescent immunoassay, and flow cytometry, except for anti-Ro52/TRIM21, anti-Ro60, and ANA, whose assays are described below. Anti-Ro52/TRIM21, anti-SSA, and anti-SSB antibodies were detected by line immunoassay using immunoblot strips (Shanghai Kexin Biotech Co., Ltd., China) and reported as negative, +, ++, or +++ (semi-quantitative grading of band colour intensity in arbitrary signal-intensity units; +, 11–25; ++, 26–50; +++, ≥51). Antinuclear antibody (ANA) was assessed by indirect immunofluorescence on HEp-2 cells (positive at a titer ≥1:40). Myositis-specific antibodies (anti-MDA5 and anti-synthetase [Jo-1, PL-7, PL-12, EJ, OJ]) were detected by EUROLINE immunoblot assay (Euroimmun, Lübeck, Germany) on an automated EUROBlotMaster system and reported as negative, +, ++, or +++ (semi-quantitative grading of band colour intensity relative to the control band). The same assay platforms and grading thresholds were used consistently throughout the 2010–2020 study period. ILD confirmation required bilateral parenchymal infiltrates on HRCT (22), reviewed by two pulmonary rheumatologists and validated by a thoracic radiologist. A pulmonologist classified radiologic patterns as UIP, NSIP, organizing pneumonia (OP), lymphoid interstitial pneumonia (LIP), or unclassifiable. PFTs included total lung capacity, forced vital capacity, and diffusing capacity for carbon monoxide (percentage of predicted; abnormal if <80% or <70%, respectively). Overlapping HRCT patterns (e.g., UIP+NSIP) were assigned to the non-NSIP component by a pre-specified hierarchical rule (UIP+NSIP→UIP, OP+NSIP→OP, LIP+NSIP→LIP). CTD and IIM subtypes were classified according to current classification criteria: idiopathic inflammatory myopathy per the 2017 EULAR/ACR criteria (23), with dermatomyositis and polymyositis classified per the same criteria and clinically amyopathic dermatomyositis [CADM] defined clinically (characteristic cutaneous manifestations of dermatomyositis without clinical muscle weakness); anti-synthetase syndrome defined by aminoacyl-tRNA synthetase antibody positivity with compatible clinical features, informed by published preliminary criteria (24, 25); primary Sjögren’s syndrome per the 2016 ACR/EULAR criteria (26); systemic lupus erythematosus per the 2019 EULAR/ACR criteria (27); rheumatoid arthritis per the 2010 ACR/EULAR criteria (28); systemic sclerosis per the 2013 ACR/EULAR criteria (29); and ANCA-associated vasculitis per the 2022 ACR/EULAR criteria (30). Myositis-specific antibodies were assessed in a subset of patients who underwent myositis-specific antibody testing (*n* = 375; not restricted to patients with a myositis diagnosis), using the EUROLINE myositis antibody panel described above, and were used in interaction analyses (anti-MDA5 and anti-synthetase antibodies, the latter defined as reactivity to any of Jo-1, PL-7, PL-12, EJ, or OJ and combined as the ARS variable; **Supplementary Figure S1**; **Supplementary Table S1**). Among anti-MDA5-positive patients in this subset, the large majority had a dermatomyositis-spectrum phenotype (dermatomyositis ~69%, classified per the 2017 EULAR/ACR criteria; clinically amyopathic dermatomyositis ~26%, defined clinically), with overlapping CTD in ~6%, consistent with the established disease association of anti-MDA5. The study was approved by the Human Research Ethics Committee of the Second Affiliated Hospital of Zhejiang University School of Medicine (Approval No. 2020–840 of the institutional review board) in accordance with the Declaration of Helsinki, and the requirement for informed consent was waived because of the retrospective design and use of anonymized data.

### Follow-up

Eligible patients were identified among adults discharged from the study hospital between 1 August 2010 and 1 August 2020; the ascertainment window was intentionally restricted to admissions ending before 1 August 2020 (predominantly pre-pandemic) to avoid COVID-19–related period effects on ILD presentation, treatment, and prognosis. Follow-up through May 2023 documented 181 deaths (34%, verified with family members), 226 survivors (42%), and 131 patients lost to follow-up [24%; 119 (90.8%) telephone contact failure, 2 (1.5%) refused, 10 (7.6%) undocumented]. Based on the last hospitalization records, most deaths were attributed to progressive ILD or respiratory failure. Because cause of death was not uniformly recorded, all deaths were analyzed as all-cause events. Median follow-up was 1,542 days (approximately 50.7 months) by the reverse Kaplan–Meier method (SE 25.30 days; 95% CI 1486–1584 days).

### Statistical analysis

Missing values in FVC% and DLCO% (FVC 41%, DLCO 49%) were addressed by multiple imputation using chained equations (mice package; random seed 20260719). The imputation model included the survival outcome (time and status), Ro52 status, age, sex, BMI, smoking, malignancy, FVC%, FEV1%, TLC%, DLCO%, and CTD status (*m* = 20). Because the fraction of missing information was high (mean = 0.44, maximum 0.82 for DLCO%), m was increased from 5 to 20, as White et al. (31) recommend (m ≥ 20 when the fraction exceeds 0.30). Cox models were fitted in each imputed dataset and pooled by Rubin’s rules. Effect modification was examined by likelihood-ratio tests of multiplicative interaction terms (Ro52 with sex, smoking, CTD subtype, malignancy, lung function, complement, and myositis-specific antibody status).

Statistical analyses used R 4.3.2 (survival and mice packages). Continuous variables were compared by Welch’s t-test or Wilcoxon rank-sum test, categorical variables by chi-square or Fisher’s exact test, and standardized mean differences summarized balance. Survival was estimated by Kaplan–Meier, log-rank, and Cox proportional-hazards analyses. Guided by causal diagramming (backdoor criterion), the multivariable model adjusted for age, sex, BMI, smoking, malignancy, and FVC%/DLCO% predicted. Collinear autoantibodies (ANA/SS-A/Ro60/SSB) and post-baseline treatments and intensive-care interventions (corticosteroids, IVIG, ICU admission, and mechanical ventilation; ICU status was evaluated separately in sensitivity analyses, **Supplementary Table S9**) were excluded to avoid mediator and collider biases. Missing covariates used the same imputation scheme (*m* = 20), pooled by Rubin’s rules. Two-sided *P* < 0.05 denoted significance. Baseline characteristics were compared without adjustment for multiple comparisons and interpreted primarily by standardized mean differences (|SMD| > 0.1 considered clinically meaningful). For exploratory subgroup and interaction analyses, Benjamini–Hochberg false-discovery-rate-adjusted *q*-values were reported alongside unadjusted *P*-values. The Ro52×Ro60 product-interaction term was not fitted (Kaplan–Meier stratification in **Figures 2C, D** instead). A Bonferroni-corrected significance threshold (α = 0.01) was applied to the five HRCT pattern comparisons, and Fisher’s exact test was used for categorical comparisons with small expected cell counts.

**Figure 2:**
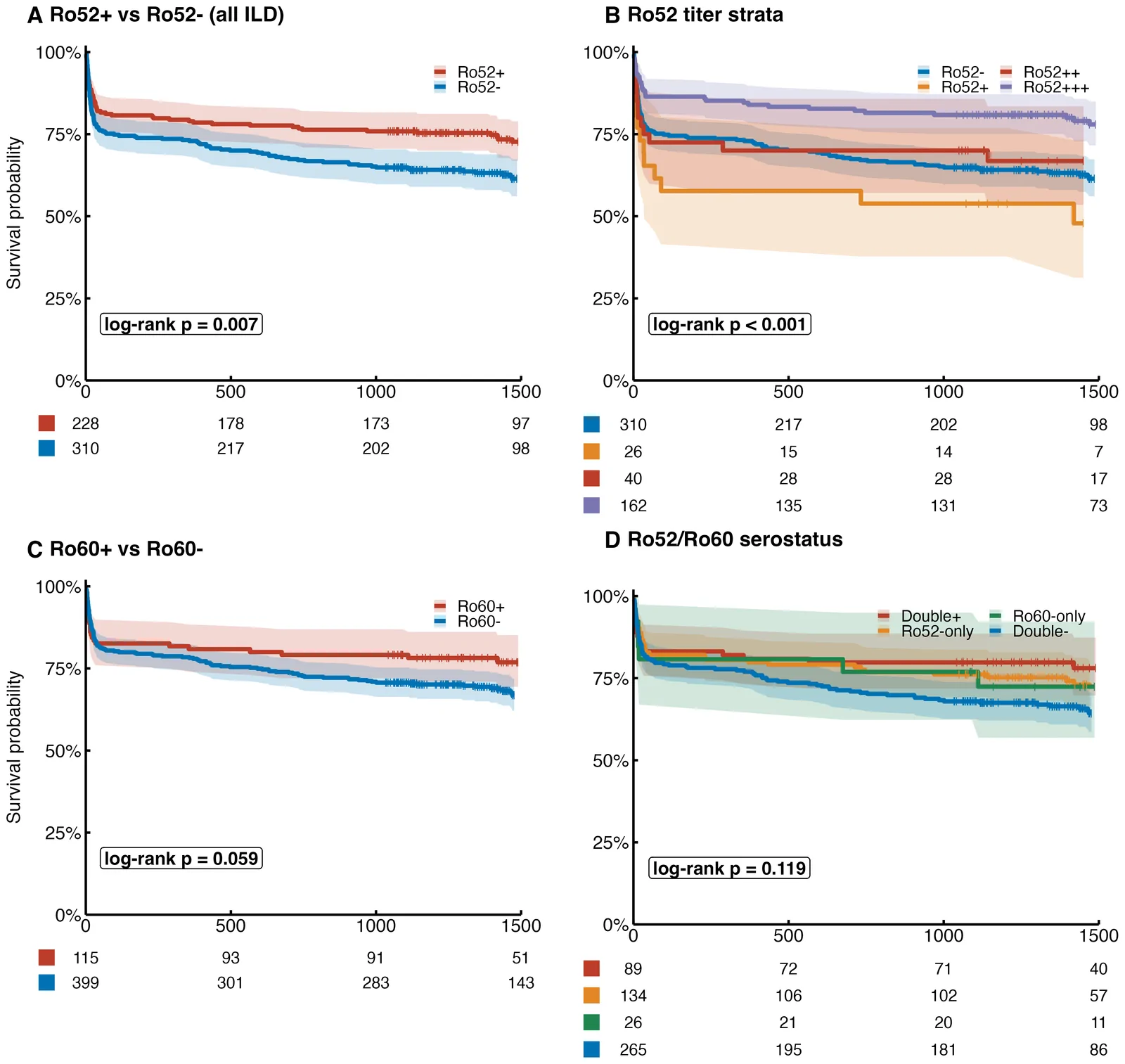
Kaplan–Meier survival curves for all-cause mortality in ILD patients. **(A)** Ro52-positive versus Ro52-negative patients (all ILD). **(B)** Ro52 titer stratification (negative, +, ++, +++). **(C)** Ro60-positive versus Ro60-negative patients. **(D)** Kaplan–Meier curves by Ro52/Ro60 serostatus combination: double-negative, Ro52-only, Ro60-only, or double-positive (24 patients with missing serostatus excluded). The reference group is anti-Ro52-negative patients; 181 deaths were observed during follow-up. Shaded bands denote 95% pointwise confidence intervals; vertical ticks denote censored observations; numbers below each panel show patients at risk at selected time points.

Sequential Cox models entered Ro52 alone (Model 1) and added age and sex (Model 2), BMI (Model 3), smoking (Model 4), malignancy (Model 5), and FVC%/DLCO% (Model 6), pooled across *m* = 20 imputations by Rubin’s rules. The pre-specified primary analysis was the fully adjusted Model 6 (C-index 0.649; range across the 20 imputed datasets 0.629–0.681). Model 7, which additionally adjusted for CTD status, served as a key sensitivity analysis for confounding by underlying diagnosis. Models 1–5 illustrate attenuation of the Ro52 effect upon successive adjustment of established confounders. The proportional-hazards assumption was examined with Schoenfeld residuals. Non-proportionality was driven by age alone (median global *P* = 0.008; Ro52 *P* = 0.235; all other covariates *P* ≥ 0.17) and was addressed with a time-varying age term [Model 6 + tt(Age); Ro52 HR 0.739, 95% CI 0.535–1.020, *P* = 0.066], which left the Ro52 estimate directionally consistent; an age-stratified analysis (median age = 60 years) likewise yielded directionally consistent Ro52 estimates in both strata (HR 0.80, 95% CI 0.50–1.28 among patients ≤ 60 years; HR 0.67, 95% CI 0.42–1.05 among patients > 60 years). Sensitivity analyses used a broader 63-case malignancy definition, a 32-case reverse-causation restriction, and inverse-probability-of-censoring weighting for the 24% loss to follow-up (IPCW-weighted Model 6: HR 0.659, 95% CI 0.405–1.072, *P* = 0.093); IPCW weights were derived from a censoring model fitted with the Model 6 covariates (Ro52 status, age, sex, BMI, smoking, malignancy, FVC%, and DLCO%), and results were essentially unchanged when CTD status was additionally included in the censoring model (HR 0.659, 95% CI 0.406–1.068, *P* = 0.091). Subgroup analyses compared CTD-ILD versus non-CTD-ILD within each Ro52 stratum using the core confounder set. The primary multiply-imputed models used all 538 patients, whereas subgroup and interaction analyses used complete-case subsets as indicated in the corresponding legends. Full Cox results for Models 1–7 are in **Table 1**.

**Table 1:** Sequential Cox proportional-hazards regression for all-cause mortality (*m* = 20 multiple imputation, Rubin’s rules).

Model	Description	Ro52 HR (95% CI)	Ro52 *P*-value	Tumor HR (95% CI)	Tumor *p*-value
Model 1	Ro52 alone	0.66 (0.48–0.90)	0.008	—	—
Model 2	+ Age + Sex	0.73 (0.53–0.99)	0.046	—	—
Model 3	+ BMI	0.73 (0.53–1.00)	0.049	—	—
Model 4	+ Smoking	0.74 (0.54–1.01)	0.059	—	—
Model 5	+ Malignancy	0.73 (0.54–1.00)	0.053	1.83 (1.17–2.87)	0.009
Model 6	+ FVC% + DLCO% (MI)	0.72 (0.52–0.99)	0.045	1.81 (1.15–2.88)	0.012
Model 7	+ CTD status	0.75 (0.54–1.04)	0.091	1.70 (1.07–2.71)	0.027

HR, hazard ratio; 95% CI, 95% confidence interval. Reference: Ro52-negative for the Ro52 HR and absence of malignancy for the Tumor HR. *P*-values from Wald tests. Models were adjusted sequentially as listed. All models were fitted in each of the *m* = 20 imputed datasets and pooled by Rubin’s rules. Model 6 additionally included FVC% and DLCO% predicted; Model 7 additionally included CTD status (vs. non-CTD-ILD), which itself was associated with lower mortality (HR 0.53, 95% CI 0.37–0.74; *P* < 0.001). Malignancy was defined as any malignancy confirmed by hospital documentation (*n* = 47/538, 8.7%). C-index (concordance) was 0.548, 0.599, 0.614, 0.616, 0.625, and 0.649 for Models 1–6, and 0.673 for Model 7, respectively (pooled across *m* = 20 imputations).

To examine whether the survival advantage of anti-Ro52 was mediated through avoidance of UIP, the radiologic pattern with the worst prognosis, we performed an exploratory counterfactual mediation analysis. The mediator (UIP vs. non-UIP) was modeled by logistic regression on anti-Ro52 status and the multivariable Cox covariate set. Outcome models with and without the mediator were fitted by Cox proportional hazards to obtain total and direct effects on the log-hazard scale. The indirect effect was computed as TE − DE, valid in the absence of an anti-Ro52 × UIP interaction (assessed by a product term, *P* = 0.080), and the proportion mediated as IE/TE. Missing FVC%/DLCO% values were addressed by median imputation (a simplification relative to the primary multiple-imputation analysis) to retain the full cohort (sensitivity against the complete-case subset in **Supplementary Table S2**). 95% confidence intervals came from 1,000 nonparametric bootstrap replicates (percentile method).

## Results

### Demographic and clinical characteristics

Among the 538 hospitalized ILD patients (mean age = 58.8 ± 13.5 years; 67.7% female, 364 of 538), 228 were Ro52-positive and 310 were Ro52-negative. Significant differences were observed in age (56.7 ± 11.7 vs. 60.3 ± 14.5 years), sex distribution (75.9% vs. 61.6% female), and admission serological markers (ANA, anti-Ro60, SSB, ALT, AST, LDH, CK, IgG, IgM, C3, C4). No differences were observed in anthropometrics (weight, BMI), smoking status, or inflammatory markers (ANCA, ferritin, IgA, lymphocytes, total T-lymphocyte count, CD4/CD8 ratio), whereas FEV1% predicted (SMD: −0.20), serum creatinine (−0.17), CRP (−0.12), and disease duration (SMD: −0.23, *P* = 0.428) showed modest imbalances (**Table 2**).

**Table 2:** Baseline characteristics of ILD patients by anti-Ro52 status.

Variable	Ro52 positive (*N* = 228)	Ro52 negative (*N* = 310)	SMD	*P*-value
Patient characteristics				
Age (years)	56.7 ± 11.7	60.3 ± 14.5	−0.27	< 0.001
Female sex	173/228 (75.9%)	191/310 (61.6%)	0.30	< 0.001
Weight (kg)	55.0 (50.0, 61.0)	56.7 (50.0, 64.4)	−0.09	0.441
BMI (kg/m2)	21.9 ± 3.1	21.9 ± 3.3	−0.01	0.941
Ever smoker	29/184 (15.8%)	46/236 (19.5%)	−0.10	0.389
CTD (vs Non-CTD)	189/228 (82.9%)	213/310 (68.7%)	0.34	< 0.001
Course of disease (days)	510 (90, 1825)	730 (120, 2555)	−0.23	0.428
ICU admission	41/228 (18.0%)	74/310 (23.9%)	−0.15	0.124
Pulmonary function tests				
FVC % predicted	76.8 ± 19.0	78.9 ± 21.1	−0.10	0.358
FEV1 (L)	1.69 (1.40, 2.09)	1.72 (1.40, 2.19)	−0.01	0.858
FEV1% predicted	76.0 ± 19.7	80.1 ± 21.2	-0.20	0.082
TLC (L)	3.56 (3.08, 4.12)	3.59 (3.07, 4.13)	−0.03	0.919
TLC % predicted	74.5 ± 13.5	74.4 ± 14.6	0.00	0.986
DLCO % predicted	51.0 ± 15.3	52.4 ± 18.4	−0.09	0.466
Laboratory findings				
ANA positive	177/223 (79.4%)	117/292 (40.1%)	0.87	< 0.001
Ro-60 positive	89/223 (39.9%)	26/291 (8.9%)	0.74	< 0.001
SSB positive	18/222 (8.1%)	2/290 (0.7%)	0.38	< 0.001
ANCA positive	3/208 (1.4%)	7/266 (2.6%)	−0.08	0.524
Alanine aminotransferase (U/L)	24 (15, 45)	19 (12, 33)	0.26	0.001
Aspartate aminotransferase (U/L)	28 (20, 57)	22 (18, 35)	0.26	< 0.001
Lactate dehydrogenase (U/L)	290 (228, 390)	246 (198, 339)	0.28	< 0.001
C-reactive protein (mg/L)	9 (4, 26)	10 (5, 30)	−0.12	0.378
Creatine kinase (U/L)	61 (36, 220)	50 (31, 77)	0.27	< 0.001
Serum creatinine (μmol/L)	52 (45, 64)	54 (46, 66)	−0.17	0.132
Ferritin (μg/L)	208 (110, 464)	164 (81, 433)	−0.00	0.263
Immunoglobulin A (g/L)	2.46 (1.83, 3.16)	2.36 (1.70, 3.37)	0.02	0.509
Immunoglobulin G (g/L)	14.3 (11.4, 17.9)	12.7 (10.3, 16.1)	0.37	< 0.001
Immunoglobulin M (g/L)	1.29 (0.84, 1.89)	1.08 (0.72, 1.61)	0.25	0.007
Complement C3 (g/L)	0.95 (0.84, 1.07)	1.01 (0.88, 1.15)	−0.30	< 0.001
Complement C4 (g/L)	0.18 (0.14, 0.22)	0.20 (0.16, 0.25)	−0.33	< 0.001
Lymphocytes (10^9/L)	1 (1, 2)	1.17 (0.79, 1.64)	0.09	0.771
Total T lymphocyte (%)	67.7 (60.4, 75.9)	71.1 (60.0, 78.8)	−0.09	0.132
CD4 to CD8 ratio	1.5 (1.0, 2.5)	1.5 (0.9, 2.3)	0.05	0.856
Malignancy	17/228 (7.5%)	30/310 (9.7%)	−0.08	0.455

Values are presented as median (IQR) or mean ± SD for continuous variables and *n* (%) for categorical variables. Ro52-positive/Ro52-negative denotes anti-Ro52 antibody serostatus. SMD, standardized mean difference. *P*-values from Welch’s t-test or Mann-Whitney U test (continuous) or chi-square/Fisher exact test (categorical). *P*-values were not adjusted for multiple comparisons and should be interpreted as descriptive. Between-group imbalance was judged primarily by standardized mean differences (|SMD| > 0.1 considered clinically meaningful). Ro-60, anti-Ro-60; SSB, anti-SS-B; ANCA, anti-neutrophil cytoplasmic antibody; ALT, alanine aminotransferase; AST, aspartate aminotransferase; LDH, lactate dehydrogenase; CRP, C-reactive protein; CK, creatine kinase; IgA/G/M, immunoglobulin A/G/M; C3/C4, complement components 3 and 4; FVC, forced vital capacity; FEV1, forced expiratory volume in 1 s; TLC, total lung capacity; DLCO, diffusing capacity for carbon monoxide. Per-variable missing-data counts are reported in **Supplementary Table S4**.

### Distribution of ILD subtypes

CTD-ILD predominated (74.7%, 402 of 538) over non-CTD-ILD (25.3%, 136 of 538) (**Supplementary Table S3**; **Supplementary Figure S2**). IIM constituted the largest CTD-ILD subset (31.0%, 167 of 538), in ascending order of frequency: PM (3.0%), CADM (3.9%), ASyS (7.6%), and DM (16.5%). Other subtypes included SSc (10.2%), RA (10.2%), SS (7.3%), SLE (5.6%), UCTD (5.0%), ANCA-associated vasculitis (3.0%), and rarer entities (e.g., MCTD, AOSD). Among the 228 Ro52-positive patients, IIM was most prevalent (43.9%), followed by non-CTD (17.1%), SS (15.4%), SSc (6.6%), and SLE (6.6%).

### Anti-Ro52 positivity across ILD subtypes

Ro52 positivity varied across ILD subtypes (**Supplementary Table S3**): highest in SS-ILD (89.7%) and MCTD-ILD (77.8%), high in CADM (76.2%), ASyS (73.2%), and IIM-ILD (59.9%), intermediate in SLE-ILD (50%) and UCTD-ILD (37.0%), and lower in non-CTD-ILD (28.7%), SSc-ILD (27.3%), RA-ILD (12.7%), and SVV (0%). CTD-ILD patients had significantly higher Ro52 positivity than non-CTD-ILD (47.0% vs. 28.7%; *P* < 0.001). Among CTD subtypes, SSc-ILD and RA-ILD were significantly lower than SS-ILD (pairwise chi-square tests, both *P* < 0.001, uncorrected for multiplicity) (**Supplementary Figure S3**).

Beyond subtype distribution, Ro52-positive patients differed substantially at baseline (**Table 2**; **Supplementary Table S4**): younger (56.7 ± 11.7 vs. 60.3 ± 14.5 years), more frequently CTD-ILD (82.9% vs. 68.7%), and less often male (24.1% vs. 38.4%; all *P* < 0.001). Consistent with an immune-mediated phenotype, they had higher creatine kinase (median 61 vs. 50 U/L), lactate dehydrogenase (290 vs. 246 U/L), transaminases, and IgG (median = 14.3 vs. 12.7 g/L), with lower complement C3 [0.95 (0.84–1.07) vs. 1.01 (0.88–1.15) g/L] and C4 [0.18 (0.14–0.22) vs. 0.20 (0.16–0.25) g/L; all *P* ≤ 0.001]. Malignancy was non-significantly less common in Ro52-positive patients (7.5% vs. 9.7%; *P* = 0.455).

### ILD radiologic patterns and Ro52 association

HRCT revealed NSIP as the dominant pattern (60.6%), followed by unclassifiable (22.5%), UIP (12.1%), OP (3.7%), and LIP (1.1%) (**Supplementary Figure S4**). Ro52-positive patients showed a distinct radiological profile: NSIP was significantly more prevalent (69.7% vs. 53.9%; *P* < 0.001), whereas UIP was substantially less frequent (4.8% vs. 17.4%; *P* < 0.001). No differences were observed in OP, LIP, or unclassifiable patterns.

### Prognostic impact of Ro52 in ILD

Univariate Cox regression revealed no significant survival association with Ro52 within individual ILD subtypes (**Supplementary Table S3**). In the pooled cohort (*n* = 538), Ro52 positivity was associated with reduced mortality in unadjusted analysis (HR 0.66, 95% CI 0.48–0.90; *P* = 0.008), confirmed by Kaplan–Meier analysis (*P* = 0.007; **Figure 2A**). By titer stratification, only high-titer Ro52 (+++) significantly lowered mortality (HR 0.50, 95% CI 0.34–0.73; *P* < 0.001), whereas Ro52+ and Ro52++ were neutral (*P* = 0.169 and *P* = 0.718; **Figure 2B**). Ro60 positivity trended toward protection (log-rank *P* = 0.059; **Figure 2C**). Overall survival did not differ across the four Ro52/Ro60 serostatus combinations (global log-rank *P* = 0.119; **Figure 2D**).

Prognosis was consistently better in CTD-ILD than in non-CTD-ILD regardless of Ro52 status: among Ro52-negative patients, non-CTD-ILD carried a 1.75-fold higher death risk (HR 1.75, 95% CI 1.20–2.57; *P* = 0.005), whereas among Ro52-positive patients, CTD-ILD showed a 58% lower risk (HR 0.42, 95% CI 0.23–0.77; *P* = 0.006). In a direct comparison, Ro52-positive CTD-ILD patients had a 65.3% lower mortality risk than Ro52-negative non-CTD-ILD patients (HR 0.35, 95% CI 0.23–0.52; *P* < 0.001; **Supplementary Figure S5A**). Hazard ratios for individual ILD subtypes are summarized in **Supplementary Table S3**. The protective association persisted after excluding DM/CADM: HR 0.61 (95% CI 0.38–0.97; *P* = 0.039) in the remaining CTD-ILD patients and HR 0.67 (95% CI 0.47–0.96; *P* = 0.031) in non-DM/CADM-ILD (adjusted for age, sex, and BMI; **Supplementary Figures S5B, C**).

An exploratory mediation analysis tested whether the Ro52→mortality association was explained by avoidance of UIP. Ro52 positivity was strongly associated with lower UIP odds (OR 0.30, 95% CI 0.12–0.56), yet UIP was not an independent predictor of mortality in the multivariable model (HR 1.27, 95% CI 0.83–1.91). The total effect of anti-Ro52 (HR 0.73, 95% CI 0.52–1.02) and the direct effect adjusted for UIP (HR 0.75, 95% CI 0.53–1.05) were nearly identical, with an indirect effect of HR 0.98 (95% CI 0.91–1.01) and a non-significant proportion mediated of 7.9% (95% CI −20.3% to 97.7%). These results indicate no mediation by UIP (**Supplementary Table S2**). The non-significant anti-Ro52 × UIP interaction (*P* = 0.080) supported the additive decomposition.

### Multivariable Cox regression analysis

In unadjusted Cox regression, Ro52 positivity was associated with 34% lower mortality (Model 1: HR 0.66, 95% CI 0.48–0.90; *P* = 0.008). Sequential adjustment for age, sex, BMI, and smoking (Model 4) attenuated the effect (HR 0.74, 95% CI 0.54–1.01; *P* = 0.059). Adding malignancy (Model 5) left the Ro52 estimate essentially unchanged (HR 0.73, 95% CI 0.54–1.00; *P* = 0.053), whereas malignancy itself was a significant predictor (HR 1.83, 95% CI 1.17–2.87; *P* = 0.009). The broader 63-case definition attenuated this association (HR 1.32, 95% CI 0.85–2.06; *P* = 0.22), whereas restricting exposure to the 32 pre-enrolment malignancies strengthened it (Model 5 HR 2.11, 95% CI 1.24–3.59; *P* = 0.006; Model 6 HR 2.18, 95% CI 1.26–3.78; *P* = 0.006). Incorporating pulmonary-function severity (Model 6) yielded HR 0.72 (95% CI 0.52–0.99; *P* = 0.045), the confidence interval just excluding 1. Further adjustment for CTD status (Model 7) attenuated the Ro52 association to non-significance (HR 0.75, 95% CI 0.54–1.04; *P* = 0.091), with CTD itself remaining strongly protective (HR 0.53, 95% CI 0.37–0.74; *P* < 0.001), consistent with Ro52 serving as a marker of the CTD phenotype rather than an independent predictor. Because CTD status is an important clinical characteristic associated with both Ro52 positivity and prognosis, Model 7, which additionally adjusts for CTD status, serves as a key sensitivity analysis for confounding by underlying diagnosis. The Model 6 estimate is therefore best regarded as a borderline finding, with Model 7 an equally compatible specification. The interpretation of anti-Ro52 as a context-dependent phenotypic marker does not depend on the statistical significance of any single model: the complete-case, time-varying-age, IPCW-weighted, and CTD-adjusted estimates were all non-significant. A delta-adjustment missing-not-at-random tipping-point analysis (**Supplementary Figure S6**) indicated the association is not an imputation artifact. Discrimination improved modestly (C-index 0.548 to 0.649), and neither the time-varying age model (HR 0.74, 95% CI 0.54–1.02; *P* = 0.066) nor inverse-probability-of-censoring weighting (HR 0.66, 95% CI 0.41–1.07; *P* = 0.093) materially altered the estimate. Ro52 is thus at most a weak independent predictor; its crude protective signal reflects the immune-mediated ILD phenotype rather than an autonomous prognostic effect (**Figure 3**). Full model coefficients are in **Table 1**.

**Figure 3:**
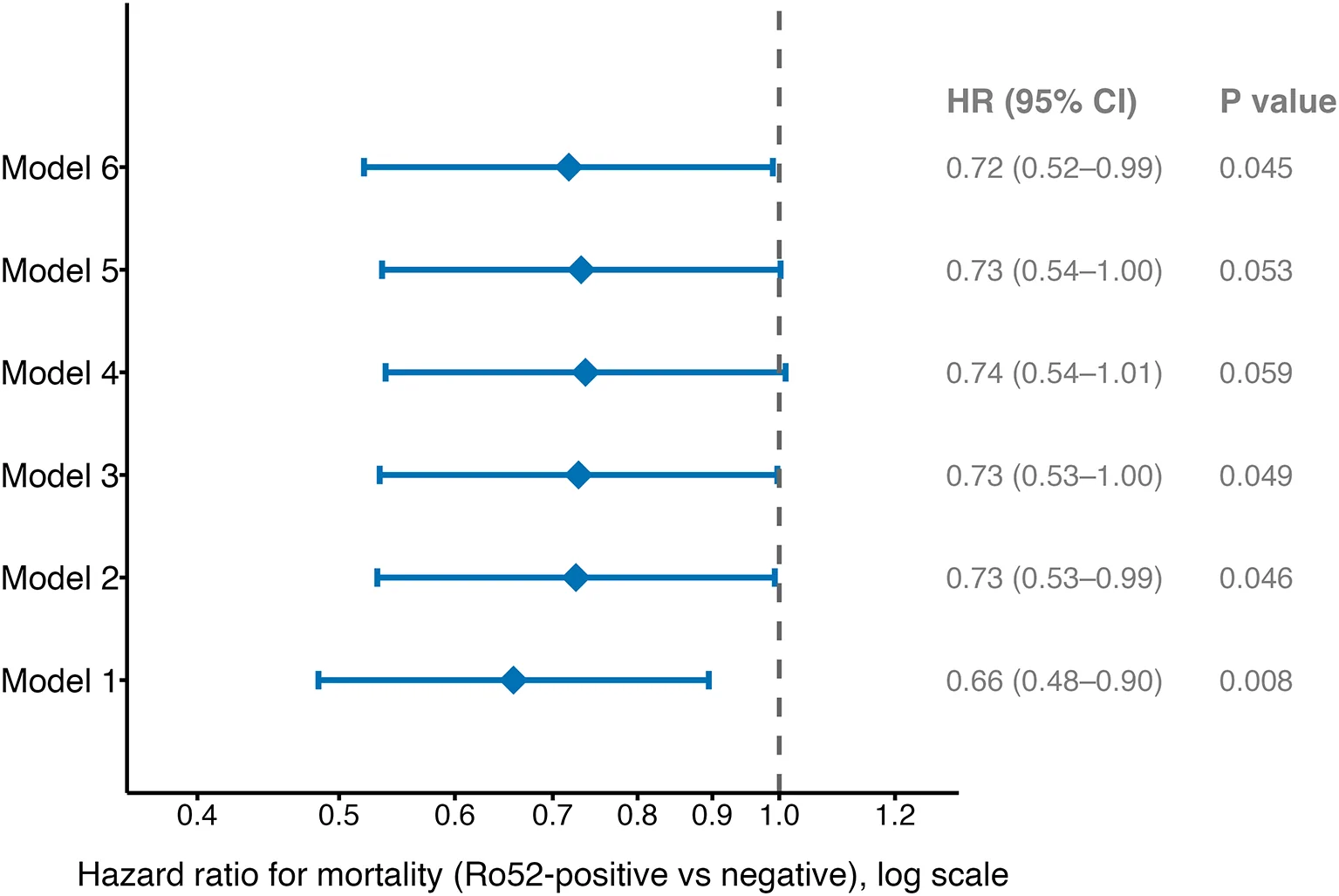
Forest plot of the Ro52 association with all-cause mortality across sequentially adjusted Cox models (Model 1: Ro52 alone. Model 2: + age + sex. Model 3: + BMI. Model 4: + smoking. Model 5: + malignancy. Model 6: + FVC% + DLCO%). All six models were fitted in each of the m = 20 multiply-imputed datasets and pooled by Rubin’s rules. The reference group is anti-Ro52-negative patients; 181 deaths were observed during follow-up. Diamond = hazard ratio; horizontal bars = 95% confidence interval; dashed line = hazard ratio = 1.0. Model 7, which additionally adjusts for CTD status, is reported in **Table 1**. Anti-Ro52 antibodies (Ro52); interstitial lung disease (ILD).

Treatment intensity tracked disease severity rather than Ro52 status per se: Ro52-positive patients received intravenous immunoglobulin (IVIG) more often (11.9% vs. 6.5%), yet IVIG use was strongly associated with worse survival overall (log-rank *P* = 0.001) and within both Ro52 strata (both *P* < 0.05). Treatments were therefore reported descriptively and used only for stratification, never as Cox covariates, to avoid mediator bias (**Supplementary Figure S7**). ICU admission and other critical-care interventions were similarly descriptive severity markers. Their distributions and survival curves are shown in **Supplementary Table S5** and **Supplementary Figure S8**.

### Effect modification and interaction analyses

Because the adjusted Ro52 effect was weak and not statistically significant in the complete-case analysis (HR 0.75, 95% CI 0.49–1.16; *P* = 0.196), we tested whether it varies by subgroup (**Figure 4**; **Supplementary Table S1**). The Ro52 × CTD-subtype interaction was significant (likelihood-ratio *P* = 0.027): Ro52 positivity trended toward lower mortality in CTD-ILD (adjusted HR 0.73, 95% CI 0.49–1.07; *P* = 0.104) but not in non-CTD disease (HR 0.90, 95% CI 0.52–1.55). The apparent protective association in men (HR 0.55, 95% CI 0.32–0.93; *P* = 0.025) did not survive false-discovery-rate adjustment (*q* = 0.069) and should be interpreted cautiously. No sex-based effect was evident in women (HR 0.88, 95% CI 0.59–1.31; *P* = 0.522). In the myositis-antibody-tested subset (*n* = 375; 372 with evaluable anti-Ro52 results), the point estimate of the anti-MDA5 effect was numerically higher among anti-Ro52-positive than Ro52-negative patients, but the formal interaction test did not reach statistical significance (likelihood-ratio *P* = 0.055), limited by the small number of events (*n* = 33 deaths in the four-combination model). Among anti-Ro52-positive patients, anti-MDA5 positivity raised mortality to 10.57 (95% CI 3.60–31.03) versus 2.56 (95% CI 0.97–6.74) among Ro52-negative patients, whereas anti-Ro52 showed no significant mortality effect within the anti-MDA5-positive subgroup (HR 1.73, 95% CI 0.66–4.54; *P* = 0.267) and no significant effect in anti-MDA5-negative disease (HR 0.44, 95% CI 0.15–1.29; *P* = 0.133). MDA5 positivity itself conferred markedly increased mortality (unadjusted HR 5.13, 95% CI 2.58–10.20), whereas anti-synthetase positivity was protective (HR 0.12, 95% CI 0.04–0.39) (**Supplementary Tables S1**, **S7**; **Supplementary Figure S1**). In a parsimonious sensitivity analysis restricted to the myositis-antibody-tested subset (*n* = 372, 33 events), adjustment for age and sex yielded a nominally significant Ro52 × MDA5 interaction (interaction HR 4.81, 95% CI 1.13–20.42; Wald *P* = 0.033; likelihood-ratio *P* = 0.030; **Supplementary Table S7B**); however, this rests on few events with a wide confidence interval and requires confirmation in larger independent cohorts.

**Figure 4:**
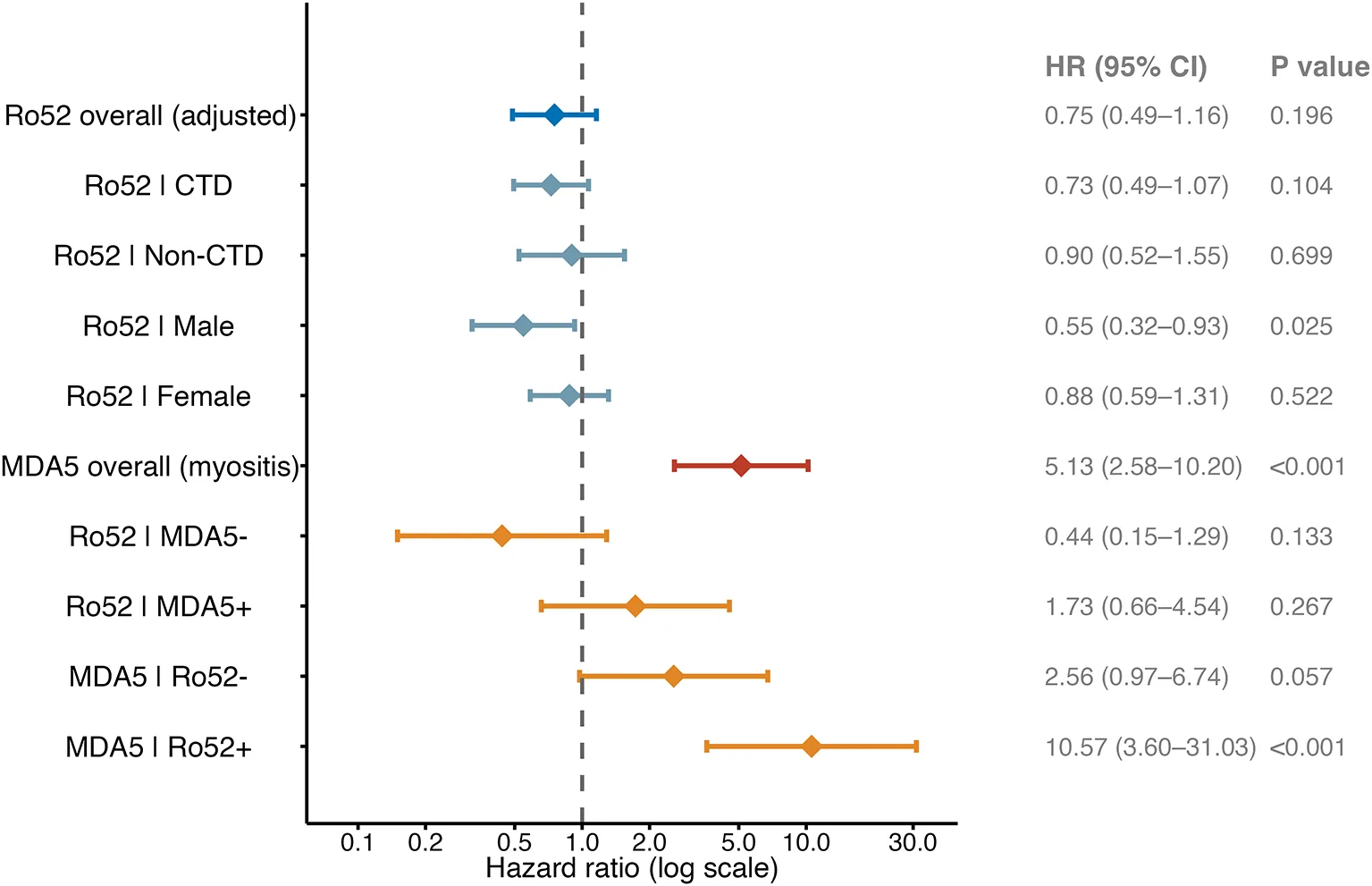
Forest plot of hazard ratios for anti-Ro52 (Ro52) and anti-MDA5 from the interaction analyses. Blue: main cohort (Ro52); orange: myositis-antibody-tested subset (MDA5/Ro52); red: overall anti-MDA5 effect in the myositis subset. In the main cohort (*n* = 538), the overall adjusted Ro52 estimate (age, sex, BMI, smoking) and estimates within CTD, non-CTD, male, and female subgroups are shown (adjusted for age, sex, and BMI as applicable). Subgroup and interaction analyses used complete-case data; the complete-case overall adjusted estimate (*n* = 420 after excluding 118 patients with missing smoking data) is shown, whereas **Table 1** reports multiply-imputed (m = 20) estimates. In the myositis-antibody-tested subset (*n* = 375), unadjusted Ro52 and MDA5 estimates are stratified by anti-MDA5 and anti-Ro52 status. Interaction *P*-values are from likelihood-ratio tests (**Supplementary Table S1**). HR, hazard ratio; CI, confidence interval; CTD, connective tissue disease; MDA5, anti-melanoma differentiation-associated protein 5; ARS, anti-synthetase. Reference groups: anti-Ro52-negative (main cohort) and anti-MDA5-negative/anti-Ro52-negative (myositis-antibody-tested subset). The myositis-antibody-tested subset comprised 375 patients with 34 deaths, of whom 372 had evaluable anti-Ro52 results and contributed 33 deaths to the four-combination model (**Supplementary Table S7A**). Subgroup *P*-values are unadjusted. Only the MDA5 overall (*q* = 0.002), ARS overall (*q* = 0.002), and MDA5 | Ro52+ (*q* = 0.002) estimates remained significant after Benjamini–Hochberg false-discovery-rate adjustment; the ARS overall estimate (HR 0.12, 95% CI 0.04–0.39) is reported in **Supplementary Table S1**.

## Discussion

In this large single-center Chinese ILD cohort with systematic anti-Ro52 testing, Ro52 positivity is a context-dependent phenotypic marker of immune-mediated ILD rather than a robust independent mortality predictor. The crude survival advantage of Ro52-positive patients was attenuated after adjustment for demographics, smoking, and lung function. It was largely explained by effect modification across CTD subtypes. These findings reframe anti-Ro52 as a serological pointer to autoimmune ILD biology and support a CTD-stratified framework for interpreting autoantibody panels in ILD prognosis.

### Molecular and immunologic basis of anti-Ro52

At the molecular level, anti-Ro52 recognizes TRIM21, a 52-kDa member of the tripartite motif (TRIM) family acting as an E3 ubiquitin ligase with roles in innate immunity and interferon-driven inflammation (32). This multifunctional identity explains why anti-Ro52 seropositivity spans diverse autoimmune and non-autoimmune contexts, from Sjögren’s syndrome and systemic lupus erythematosus to idiopathic interstitial pneumonia and certain malignancies.

Anti-Ro antibodies target a small RNA-protein complex whose main antigenic protein is a 60-kDa polypeptide (SSA). Ro52 (TRIM21) has gained attention for its clinical relevance, though the historical focus on Ro/SSA and La/SSB proteins may have hindered separate identification of Ro52/Ro60 antibodies (9, 10). Because a substantial proportion of anti-Ro52-positive ILD patients have non-CTD disease, including interstitial pneumonia with autoimmune features (IPAF) that is often misdiagnosed as IPF, anti-Ro52 testing holds considerable clinical significance (13, 17).

### Biological basis for divergent effects across ILD subtypes

The divergent prognostic associations of anti-Ro52 in CTD-ILD versus anti-MDA5-positive disease likely reflect two distinct immunologic states rather than a unitary biological effect of TRIM21 autoantibodies. In CTD-ILD, anti-Ro52 typically reflects a broader humoral autoimmune background (frequent co-positivity with ANA, anti-Ro60, and anti-SSB; **Table 2**) (13, 14, 17); such patients generally respond to immunosuppression, so the association with lower mortality reflects treatment-responsive immune-mediated disease rather than direct protection. In anti-MDA5-positive disease, by contrast, concurrent anti-Ro52 has been proposed to mark an amplified type I interferon state (MxA signaling) associated with rapidly progressive ILD (15–18); this modification reflects amplification of the adverse effect of anti-MDA5 by anti-Ro52, rather than an independent mortality effect of anti-Ro52 within the anti-MDA5-positive subgroup (HR 1.73, *P* = 0.267). Anti-Ro52 is therefore best regarded as a context-dependent marker of two distinct immunologic milieux: a comparatively indolent autoimmune state in most CTD-ILD, and a hyperactive interferon-driven state in anti-MDA5-positive disease.

### Principal findings in context of prior literature

Autoimmune diseases are more prevalent in women (33), and Ro52 positivity was more common in younger female ILD patients, consistent with existing findings (34). Although this does not necessarily explain the higher IgG expression in Ro52-positive patients, inclusion of SLE patients may confound the observed complement C3/C4 differences (17). No significant differences were observed in pulmonary function tests. Ro52-positive patients showed higher positivity for ANA, anti-Ro60, anti-SSB, and elevated ALT, AST, LDH, CK, and IgG. CTDs accounted for the majority of cases (74.7%), with IIM (including anti-synthetase syndrome) the largest proportion (31.0%), and prior studies report considerable heterogeneity in ILD prevalence across CTD subtypes (35).

Ro52 positivity varies widely across ILD subtypes in prior reports (36%–86% in SS-ILD, 8%–46% in SSc-ILD, 30% in SLE-ILD, 53%–78% in IIM-ILD) (14, 17, 36). Our RA-ILD positivity (12.7%) was the lowest in the cohort, differing significantly from SS-ILD, IIM-ILD, MCTD-ILD, and SLE-ILD (*P* < 0.001). SS-ILD showed the highest rate (89.7%), followed by IIM-ILD (59.9%) (**Supplementary Figure S3**; **Supplementary Table S3**). In purely idiopathic ILD populations, anti-Ro52 prevalence is markedly lower (37). This high prevalence in our cohort reflects CTD-ILD enrichment, with anti-Ro52 marking a continuum from overt CTD-ILD to subclinical autoimmune ILD within the IPAF spectrum.

A single-center Italian study of 60 ASyS patients reported that Ro52 status did not influence ILD radiographic pattern, lung function, or prognosis (38). By contrast, our analysis revealed a distinct radiological signature: Ro52-positive patients had significantly higher NSIP prevalence (69.7% vs. 53.9%; *P* < 0.001) and lower UIP (4.8% vs. 17.4%; *P* < 0.001), manifesting as predominant ground-glass opacities rather than honeycombing. This pattern is consistent with an immune-mediated phenotype, although treatment efficacy was not evaluated in this observational study (**Supplementary Table S5**). Consistent with prior syntheses (35), UIP predominates in RA whereas NSIP predominates in other CTD subtypes, and LIP is more common in SS. Among major CTD subtypes, RA-ILD showed the highest UIP proportion (23.6%, 13 of 55, including six UIP+NSIP patterns assigned to UIP by the hierarchical rule) and the lowest Ro52 positivity (12.7%), and UIP showed the lowest Ro52 positivity among ILD patterns (16.9%).

Existing studies on Ro52 in SSc suggest an independent association with ILD presence and poorer survival (19, 20, 39). We found no significant prognostic difference by Ro52 status in SSc-ILD (log-rank *P* = 0.248; only three deaths, all among Ro52-negative patients), likely owing to limited power (55 SSc patients, 15 Ro52-positive) and disease heterogeneity. Ro52 likewise had no significant impact in CTD-ILD overall (*P* = 0.104, HR 0.73, 95% CI 0.49–1.07), consistent with a single-center study of 238 CTD-ILD patients (40). Ro52-positive CTD-ILD patients nevertheless had a 65.3% lower mortality risk than Ro52-negative non-CTD-ILD (HR 0.35, 95% CI 0.23–0.52; *P* < 0.001; **Supplementary Figure S5A**). This suggests Ro52 reflects a broader immune-mediated CTD phenotype, although the within-CTD association was not statistically significant (*P* = 0.104).

Recent studies (41, 42) linked Ro52 positivity to higher mortality in anti-MDA5-ILD patients. Our findings diverge: we suggest a lower-mortality association of Ro52 in specific ILD subtypes, particularly across the non-DM/CADM spectrum (HR 0.67, 95% CI 0.47–0.96; *P* = 0.031), while the within-CTD association was not statistically significant (*P* = 0.104). This indicates context-dependent effects even among distinct CTD entities. Xu et al. and Gui et al. (43, 44) likewise reported poor prognosis with Ro52 positivity in anti-MDA5 dermatomyositis.

### Concomitant malignancy

Ro52-positive ILD is enriched for CTD-ILD and depleted of malignancy (7.5% vs. 9.7%; *P* = 0.455). In our imputed models, malignancy was an independent predictor of mortality in the primary analysis restricted to the 47 hospital-documented cases (**Table 1**), but the Ro52 effect did not change materially when malignancy was added. This indicates confounding by disease phenotype rather than by malignancy per se. The association was attenuated under the broader 63-case definition (likely false-positive flags) yet strengthened in the 32 pre-enrolment-malignancy analysis, excluding reverse causation from incident cancers. The apparent “protective” association of Ro52 therefore largely reflects its linkage to a less aggressive disease constellation rather than direct biological protection.

### Effect modification reconciling protective and adverse signals

In the largest cohort reported to date (1,026 ILD patients tested for anti-Ro52), Imai and colleagues found that anti-Ro52 seropositivity was associated with higher risk of ILD progression or all-cause death (HR 2.10; 95% CI 1.61–2.73) and of lung transplantation or death (HR 1.61; 95% CI 1.11–2.35) on multivariable analysis (45–47). This contrasts with the crude protective association in our cohort. Imai et al. defended their composite-outcome analysis in their response (46) to the accompanying commentary (47). The discrepancy most plausibly reflects differences in outcome definition and cohort composition (46, 47). The prognostic effect of Ro52 is thus modified not only by ILD subtype and autoantibody profile, but also by the choice of outcome measure.

Anti-Ro52 appears protective in our cohort but adverse in others because of effect modification. In the full cohort, Ro52 positivity tended toward lower mortality in CTD-ILD, with no robust sex-specific effect after false-discovery-rate adjustment (men: *P* = 0.025, *q* = 0.069; women: *P* = 0.522) and no effect in non-CTD disease. The contrast was numerically most pronounced in the myositis-antibody-tested subset, where anti-MDA5 positivity raised the mortality hazard to 10.57-fold among anti-Ro52-positive patients versus 2.56-fold among Ro52-negative patients (interaction *P* = 0.055), whereas anti-Ro52 itself showed no significant effect within the anti-MDA5-positive subgroup (HR 1.73, *P* = 0.267). This fits reports that anti-Ro52 coexistence identifies rapidly progressive ILD and death in anti-MDA5 dermatomyositis (48) and that anti-Ro52 positivity tracks mortality there (41, 42). Cohorts with a broader ILD case mix and composite progression endpoints, such as Imai’s (45), therefore report an adverse association for anti-Ro52, whereas our broader sample shows a net lower-mortality association because most patients have non-MDA5 CTD, in which anti-Ro52 was not associated with increased mortality. Anti-Ro52 is thus best understood as a context-dependent phenotypic marker rather than a standalone prognostic biomarker, as supported by an exploratory mediation analysis (proportion mediated 7.9%, 95% CI −20.3%–97.7%): the survival advantage reflects immune-mediated disease biology not captured by HRCT pattern alone.

### Clinical implications

These findings translate into CTD-stratified risk stratification and monitoring rather than therapeutic recommendations, because this observational study evaluates mortality associations and not treatment efficacy. First, in CTD-ILD (the largest subset, 74.7%), anti-Ro52 positivity reflected a broader immune-mediated phenotype and showed a trend toward lower mortality that was not statistically significant (HR 0.73, 95% CI 0.49–1.07, *P* = 0.104). Second, among patients with myositis-spectrum ILD, anti-MDA5 positivity identifies the highest-risk subgroup, and the adverse prognostic effect of anti-MDA5 was numerically greater in the presence of anti-Ro52 (MDA5 HR 10.57, 95% CI 3.60–31.03 among Ro52-positive vs. 2.56, 95% CI 0.97–6.74 among Ro52-negative patients), although this interaction did not reach statistical significance (*P* = 0.055); anti-Ro52 itself did not significantly increase mortality within the anti-MDA5-positive subgroup (HR 1.73, 95% CI 0.66–4.54). Such patients warrant close monitoring of respiratory status, including serial FVC, pulse oximetry, and symptom surveillance. Third, in patients without overt CTD features, HRCT UIP pattern and progressive FVC decline rather than Ro52 status itself should drive prognostic counselling. These considerations embed autoantibody panels within disease-subtype stratification, positioning anti-Ro52 as a CTD-contextual modifier of ILD prognosis rather than a basis for therapeutic decisions.

## Strengths and limitations

This study benefits from a large, well-characterized ILD cohort (*n* = 538) with detailed phenotyping and extended follow-up (median approximately 50.7 months). Limitations include the retrospective single-center design, a 24.3% loss-to-follow-up rate, and unmeasured confounders, as reflected by the attenuation of the hazard ratio from 0.66 (unadjusted) to 0.72 (fully adjusted). The sampling window was restricted to admissions ending before 1 August 2020 (predominantly pre-pandemic), to avoid COVID-19–related period effects on ILD outcomes; although this limits the currency of the data, known survival status in 407 of 538 patients (75.7%), including 181 deaths, ensured sufficient event maturity. Follow-up briefly crossed the first wave of COVID-19 in Zhejiang Province (December 2022 to January 2023). Only 10 of 181 deaths (5.5%) occurred after December 2022, including 4 (2.2%) during the January 2023 peak, and their distribution was balanced across Ro52 groups [3 of 10 (30%) Ro52-positive, comparable to the 34.3% proportion among all decedents]; even under the most extreme assumption that all were pandemic-attributable, their impact on the Ro52-mortality association would be negligible. In addition, pandemic exposure is not known to be associated with Ro52 serostatus, so any such deaths would enter the all-cause endpoint non-differentially and would not bias the estimate. Because Ro52 testing was performed at clinicians’ discretion, selection bias cannot be excluded. Additionally, preferentially selecting the ICU record as the index hospitalization for patients with multiple admissions is a potential source of selection bias. Records were assembled in successive extraction waves to complete missing data, including a later wave that contributed 81 ICU-admitted patients to the final cohort, all applying identical outcome-independent eligibility criteria. Sensitivity analyses addressing ICU-related selection showed that adjustment for ICU status attenuated the estimate to the null (HR 0.91, 95% CI 0.65–1.28, *P* = 0.594), consistent with ICU admission capturing illness severity and case mix, while exclusion of ICU admissions preserved the direction of the association without reaching significance (HR 0.59, 95% CI 0.35–1.01, *P* = 0.057), consistent with limited statistical power in this smaller subset (**Supplementary Table S9**); neither analysis altered the interpretation of anti-Ro52 as a context-dependent marker. Among otherwise eligible ILD patients captured in the data-extraction tables but not enrolled (*n* = 126), detailed baseline data were not abstracted; however, the proportion with a CTD diagnosis was lower than in the enrolled cohort (60.3% vs. 74.7%), suggesting that discretionary testing modestly enriched the cohort for CTD-ILD; this direction would bias the crude association toward protection rather than toward an adverse effect, and our interpretation of anti-Ro52 as a context-dependent marker does not depend on the magnitude of the protective association. Comprehensive myositis/ENA antibody panels were not uniformly available, limiting our ability to disentangle Ro52 from MDA5, Scl-70, and other prognostic autoantibodies. Pulmonary hypertension and patient-reported dyspnoea or functional status were likewise not systematically captured, representing additional unmeasured prognostic dimensions. Cause of death, including acute exacerbation-related deaths, was not uniformly recorded, precluding a formal competing-risk (Fine-Gray) analysis. We therefore used all-cause mortality as a pragmatic proxy for ILD-related mortality, supported by a review of the last hospitalization of the 181 decedents (134 [74.0%] discharged within 90 days of death; leading terminal diagnoses ILD, dermatomyositis, SLE, and interstitial pneumonia). A loss-to-follow-up sensitivity analysis (24.3% lost) indicated stability of the Ro52-mortality association, with telephone contact failure the predominant reason (90.8%), supporting non-differential censoring; Ro52 positivity was similar in lost versus retained patients (41.2% vs. 42.8%, *P* = 0.836) (**Supplementary Table S8**). The pre-specified IPCW analysis likewise crossed the null (HR 0.66, 95% CI 0.41–1.07, *P* = 0.093), consistent with the other robustness analyses. Lost patients were more frequently CTD-ILD than retained patients (84.0% vs. 71.7%, *P* = 0.007); because CTD-ILD carries a better prognosis, differential loss of CTD patients would bias the cohort toward higher mortality rather than create a spurious Ro52 association, and adding CTD status to the IPCW censoring model left the estimate essentially unchanged (HR 0.659, 95% CI 0.406–1.068, *P* = 0.091). The findings suggest clinical utility for phenotype characterization in NSIP-predominant ILD but do not establish Ro52 as an independent prognostic biomarker. Validation in prospective multicenter studies using different assay platforms and serial antibody measurements is needed. In the survival-by-disease analysis (**Supplementary Table S6**, **Supplementary Figure S9**), a few entities were sparsely represented (e.g., AAV, *n* = 16, 12 deaths), so their hazard ratios are imprecise. DLCO% was missing in 49% of the cohort (FVC 41%) and may not be missing at random. Although multiple imputation under a missing-at-random assumption was primary, a delta-adjustment sensitivity analysis showed the protective Ro52 effect in Model 6 remained stable (pooled HR ~0.69–0.70, *P* < 0.05) (**Supplementary Figure S6**). Baseline characteristics and subgroup analyses involved multiple univariate comparisons without formal multiplicity adjustment. Accordingly, *P*-values in **Table 2** and per-stratum estimates in **Supplementary Table S1** are descriptive, and effect modification was evaluated by likelihood-ratio interaction tests rather than per-stratum *P*-values. The 95% confidence interval around the fully adjusted hazard ratio (0.72, 95% CI 0.52–0.99) was wide, indicating limited precision; the study was adequately powered to exclude a strong independent prognostic effect but underpowered to confirm a weak one (a *post-hoc* assessment), reinforcing the interpretation of Ro52 as a weak, context-dependent phenotypic marker.

## Conclusion

Anti-Ro52 seropositivity is a context-dependent phenotypic marker of immune-mediated ILD, not a standalone prognostic biomarker. Its prognostic association is heterogeneous across ILD phenotypes rather than uniformly protective or adverse: the within-CTD association was not statistically robust (adjusted HR 0.73, 95% CI 0.49–1.07, *P* = 0.104), whereas the adverse prognostic effect of anti-MDA5 was numerically greater among anti-Ro52-positive than Ro52-negative patients (MDA5 HR 10.57 vs. 2.56), although this interaction did not reach statistical significance (*P* = 0.055). These findings help reconcile the apparent contradiction across published cohorts and support risk stratification rather than therapeutic recommendations: in anti-MDA5-positive patients, concurrent anti-Ro52 identifies a subgroup warranting intensive respiratory monitoring, whereas in CTD-ILD Ro52 status should not be interpreted in isolation. Ro52 serostatus should therefore be interpreted within the autoantibody and disease-subtype context, pending prospective validation with serial antibody measurements.

## Data Availability

The datasets presented in this article are not readily available because they contain clinical information and are subject to patient privacy considerations. Requests to access de-identified data should be directed to the corresponding authors, Jing Xue (jingxue@zju.edu.cn) and Man Huang (huangman@zju.edu.cn), and will be considered on reasonable request subject to a data-transfer agreement. The analysis code is also available from the corresponding authors on reasonable request.

## Supplementary material

Supplementary Figure 1 Kaplan–Meier survival curves for the myositis-antibody-tested subset (*n* = 375): by anti-MDA5 status, anti-synthetase (ARS) status, anti-Ro52 status, and any myositis-antibody positivity. Shaded bands denote 95% pointwise confidence intervals; tick marks indicate censoring; log-rank *P*-values are shown within each panel. The anti-Ro52 panel includes 372 patients with evaluable anti-Ro52 results.

Supplementary Figure 2 Distribution of ILD subtypes in the overall ILD cohort (*n* = 538).

Supplementary Figure 3 Anti-Ro52 positivity proportions across individual ILD diagnoses. Descriptive analysis; no formal significance testing was performed.

Supplementary Figure 4 Distribution of high-resolution CT (HRCT) patterns among 538 ILD patients overall **(A)** and stratified by anti-Ro52 status **(B)**, after hierarchical assignment of mixed patterns (e.g., UIP+NSIP) to the non-NSIP component (UIP+NSIP→UIP, OP+NSIP→OP, LIP+NSIP→LIP). *P*-values for the Ro52-stratified comparison (B, Pearson chi-square except Fisher exact for LIP): NSIP *P* < 0.001; unclassifiable *P* = 0.128; UIP *P* < 0.001; OP *P* = 0.809; LIP *P* = 0.088. NSIP, non-specific interstitial pneumonia; UIP, usual interstitial pneumonia; OP, organizing pneumonia; LIP, lymphoid interstitial pneumonia.

Supplementary Figure 5 Kaplan–Meier survival curves by disease subgroup. **(A)** Ro52-positive CTD-ILD versus Ro52-negative non-CTD-ILD. **(B)** Non-DM/CADM ILD patients with Ro52-positive versus Ro52-negative status. **(C)** Non-DM/CADM CTD-ILD patients with Ro52-positive versus Ro52-negative status. Shaded areas indicate 95% confidence intervals; vertical ticks denote censored observations; numbers below each panel show patients at risk at selected time points.

Supplementary Figure 6 MNAR Δ-adjustment tipping-point sensitivity analysis. Ro52 remained protective (pooled HR ~0.69–0.70, *P* < 0.05) for all Δ from 0 to 20% predicted, and the tipping point was not reached within this range. Dashed and dotted lines indicate the null hazard ratio (HR = 1) and the missing-at-random (MAR) baseline estimate, respectively.

Supplementary Figure 7 Overall survival stratified by intravenous immunoglobulin (IVIG) use, in the overall cohort and within Ro52-positive and Ro52-negative subgroups (log-rank *P* = 0.001, 0.022, and 0.006, respectively). IVIG status was documented in 464 of 538 patients (26 Ro52-positive and 48 Ro52-negative patients had missing IVIG status and were excluded from this analysis). IVIG use tracked disease severity rather than Ro52 status per se. Shaded bands denote 95% pointwise confidence intervals; tick marks indicate censoring; numbers below each panel show patients at risk at selected time points.

Supplementary Figure 8 Kaplan–Meier survival stratified by intensive-care-unit (ICU) admission in the overall cohort **(A)**, Ro52-positive patients **(B)**, and Ro52-negative patients **(C)**. ICU admission is presented as a descriptive marker of illness severity, not as a Cox-model covariate. All three panels showed strong associations between ICU admission and mortality (log-rank *P* < 0.001 for each). Shaded bands denote 95% pointwise confidence intervals; tick marks indicate censoring.

Supplementary Figure 9 Kaplan–Meier all-cause survival curves by underlying disease entity (*n* = 538). The upper panel shows high-survival entities in which median overall survival was not reached (IIM, RA, SSc, SS, UCTD, MCTD); the lower panel shows lower-survival entities in which median overall survival was reached (Non-CTD, SLE, AAV). Groups with *n* ≥ 5 are shown; within-panel log-rank *P* < 0.001 (panel A) and *P* = 0.395 (panel B); global log-rank *P* < 0.001. SVV (*n* = 3) and AOSD (*n* = 1) are omitted from the plot (see **Supplementary Table S6**).

## Glossary

ILD: Interstitial Lung Disease
IPF: Idiopathic Pulmonary Fibrosis
SS-A: Sjögren’s syndrome A antigen
ARS: Anti-synthetase
MxA: Myxovirus resistance protein A
ENA: Extractable nuclear antigen
Ro52: Anti-Ro52 antibody
BMI: Body Mass Index
FVC: Forced Vital Capacity
FEV1: Forced Expiratory Volume in 1 second
TLC: Total Lung Capacity
DLCO: Diffusing capacity for carbon monoxide
PFT: Pulmonary function tests
ANA: Antinuclear antibody
ANCA: Antineutrophil cytoplasmic antibody
Ro60: Anti-Ro60 antibody
SSB: Sjögren’s syndrome B antibody
ALT: Alanine Aminotransferase
LDH: Lactate Dehydrogenase
AST: Aspartate Aminotransferase
CRP: C-Reactive Protein
CK: Creatine Kinase
Scr: Serum Creatinine
IgA: Immunoglobulin A
IgG: Immunoglobulin G
IgM: Immunoglobulin M
C3: Complement Component 3
C4: Complement Component 4
CD4: Cluster of Differentiation 4
CD8: Cluster of Differentiation 8
MCTD: Mixed Connective Tissue Disease
UCTD: Undifferentiated Connective Tissue Disease
SLE: Systemic Lupus Erythematosus
SS: Sjögren’s syndrome
RA: Rheumatoid Arthritis
SSc: Systemic Sclerosis
non-CTD: Non-Connective Tissue Disease
IIM: Idiopathic Inflammatory Myopathy
NSIP: Non-Specific Interstitial Pneumonia
UIP: Usual Interstitial Pneumonia
OP: Organizing Pneumonia
LIP: Lymphoid Interstitial Pneumonia
IPAF: Interstitial pneumonia with autoimmune features
DM/CADM: Dermatomyositis/clinically amyopathic dermatomyositis
Unclassifiable: Unclassifiable Interstitial Lung Disease
CTD: Connective Tissue Disease
HRCT: High-resolution computed tomography
IVIG: Intravenous immunoglobulin
ICU: Intensive Care Unit
EMRS: Electronic medical record system
PM: Polymyositis
ASyS: Anti-synthetase syndrome
AAV: ANCA-associated vasculitis
SVV: Small-vessel vasculitis
AOSD: Adult-onset Still’s disease
MDA5: Melanoma differentiation-associated protein 5
ECMO: Extracorporeal membrane oxygenation
HR: Hazard ratio
CI: Confidence interval
SMD: Standardized mean difference
SE: Standard error
MNAR: Missing Not At Random
IPCW: Inverse Probability of Censoring Weighting
FDR: False Discovery Rate
MI: Multiple Imputation
TRIM: Tripartite Motif
ELISA: Enzyme-Linked Immunosorbent Assay
EULAR: European Alliance of Associations for Rheumatology
ACR: American College of Rheumatology
