# Supplementary material for: Anti-Ro52/TRIM21 seropositivity in interstitial lung disease: effect modification by disease subtype and myositis-specific antibody context
Source: Front Immunol. 2026 Oct 16;17:1960409. doi: 10.3389/fimmu.2026.1960409 (PMC13719467; doi:10.3389/fimmu.2026.1960409)
Supplement: Supplementary file 1 [file DataSheet1.zip › Supplementary_Tables.docx]

**Supplementary tables**

Supplementary Tables S1-S9 supporting the manuscript "Anti-Ro52/TRIM21 seropositivity in interstitial lung disease: effect modification by disease subtype and myositis-specific antibody context". Tables are cited in the main text and provided here as a single supplementary file.

Table S1. Effect modification and interaction analyses for anti-Ro52 (Ro52) and all-cause mortality

| **Label** | **Stratum** | **N** | **HR (95% CI)** | **P-value** | **q (FDR)** |
| --- | --- | --- | --- | --- | --- |
| Ro52 overall (adjusted) | Main | 420 | 0.75 (0.49–1.16) | 0.196 | 0.270 |
| Ro52 \| CTD | Main: by CTD | 402 | 0.73 (0.49–1.07) | 0.104 | 0.191 |
| Ro52 \| Non-CTD | Main: by CTD | 136 | 0.90 (0.52–1.55) | 0.699 | 0.699 |
| Ro52 \| Male | Main: by Sex | 174 | 0.55 (0.32–0.93) | 0.025 | 0.069 |
| Ro52 \| Female | Main: by Sex | 364 | 0.88 (0.59–1.31) | 0.522 | 0.574 |
| MDA5 overall (myositis) | Myositis | 375 | 5.13 (2.58–10.20) | <0.001 | 0.002 |
| ARS overall (myositis) | Myositis | 375 | 0.12 (0.04–0.39) | <0.001 | 0.002 |
| Ro52 \| MDA5- | Myositis: by MDA5 | 297 | 0.44 (0.15–1.29) | 0.133 | 0.209 |
| Ro52 \| MDA5+ | Myositis: by MDA5 | 75 | 1.73 (0.66–4.54) | 0.267 | 0.326 |
| MDA5 \| Ro52- | Myositis: by Ro52 | 173 | 2.56 (0.97–6.74) | 0.057 | 0.125 |
| MDA5 \| Ro52+ | Myositis: by Ro52 | 199 | 10.57 (3.60–31.03) | <0.001 | 0.002 |

Stratified Cox models were fitted on complete-case data; the overall adjusted Ro52 estimate (n = 420 after excluding 118 patients with missing smoking data) is from the interaction model; multiply-imputed (m = 20) estimates are in Table 2. HR, hazard ratio; 95% CI, 95% confidence interval; P, Wald test P-value. P values in the stratified table are unadjusted; Benjamini–Hochberg false-discovery-rate-adjusted q values are provided in the final column. Pre-specified primary interactions are Ro52 × CTD, Ro52 × Sex, and (myositis subset) MDA5 × Ro52 / Ro52 × ARS. MDA5, anti-MDA5; ARS, anti-synthetase; CTD, connective tissue disease.

**Table S1 (continued). Likelihood-ratio test P-values for Ro52 × covariate interactions**

| **Interaction** | **LRT P-value** | **q (FDR)** | **Note** |
| --- | --- | --- | --- |
| Main: Ro52 x CTD | 0.027 | 0.039 | robust |
| Main: Ro52 x Sex | 0.141 | 0.156 | ns |
| Main: Ro52 x Smoke* | <0.001 | <0.001 | separation-inflated |
| Main: Ro52 x Tumor* | <0.001 | <0.001 | separation-inflated |
| Main: Ro52 x FVC%* | <0.001 | <0.001 | exploratory |
| Main: Ro52 x DLCO%* | <0.001 | <0.001 | exploratory |
| Main: Ro52 x C3* | <0.001 | <0.001 | exploratory |
| Main: Ro52 x C4* | <0.001 | <0.001 | exploratory |
| Myositis: Ro52 x MDA5 | 0.055 | 0.069 | ns (large effect, limited events) |
| Myositis: Ro52 x ARS | 0.397 | 0.397 | ns |

Interactions marked * are exploratory. Benjamini–Hochberg false-discovery-rate-adjusted q values (final column) were computed across the 10 likelihood-ratio tests in this table. Continuous covariates with separation-inflated likelihood-ratio test P-values (e.g., Ro52 × FVC%, DLCO%, C3, C4, smoking, malignancy) reflect quasi-complete separation and are not interpretable as conventional P-values; for these interactions only the direction of association and the hazard ratio are reported. The pre-specified primary interactions are Ro52 × CTD, Ro52 × Sex, and (myositis subset) MDA5 × Ro52 / Ro52 × ARS.

Table S2. Exploratory mediation analysis of anti-Ro52 positivity on all-cause mortality, mediated by HRCT UIP pattern (n = 538)

| **Path / effect** | **Estimate (log-scale)** | **HR / OR** | **95% CI** | **P-value** |
| --- | --- | --- | --- | --- |
| Ro52 → UIP (mediator model, logit) | -1.21 | OR 0.30 | 0.12–0.56 | P < 0.001 |
| UIP → death (outcome model, Cox, adj. for Ro52+covariates) | 0.24 | HR 1.27 | 0.83–1.91 | P = 0.236 |
| Total effect — Ro52 → death (no mediator) | -0.31 | HR 0.73 | 0.52–1.02 | P = 0.060 |
| Direct effect — Ro52 → death (adj. for UIP) | -0.29 | HR 0.75 | 0.53–1.05 | P = 0.104 |
| Indirect effect via UIP | -0.024 | HR 0.98 | 0.91–1.01 | P = 0.242 |
| Proportion mediated (log scale) | 7.9% | — | -20.3% to 97.7% | P = 0.290 |
| Ro52 × UIP interaction (product term) | P = 0.080 | — | — | P = 0.080 |

Method: Counterfactual mediation with Cox outcome. Mediator model: logistic regression of UIP on anti-Ro52 + age + sex + BMI + smoking + FVC% + DLCO%. Outcome models: Cox proportional hazards with and without the mediator (TE and DE on log-HR scale). Indirect effect = TE − DE (valid under no Ro52 × UIP interaction, assessed by a product term, P = 0.080). Proportion mediated = IE/TE. Bootstrap 95% CIs from 1000 nonparametric replicates (percentile method).

Abbreviations: OR, odds ratio (mediator model); HR, hazard ratio (Cox model); TE, total effect; DE, direct effect (adjusted for mediator); IE, indirect effect via UIP. FVC% and DLCO% missing values were handled by median imputation to retain the full cohort; the complete-case subset (N = 273 with 37 events, biased toward survivors) yielded qualitatively similar results (TE HR 0.45 vs DE HR 0.46, IE HR 0.98, proportion mediated 2.4%) but is reported here only for transparency.

Table S3. Baseline distribution of 538 ILD patients across 14 disease categories and anti-Ro52 positivity / all-cause mortality by disease

| **Disease** | **N** | **Ro52-positive (n)** | **Ro52-positive (%)** | **Events (Ro52+)** | **Events (Ro52-)** | **HR (95% CI)** | **P-value** |
| --- | --- | --- | --- | --- | --- | --- | --- |
| Non-CTD | 136 | 39 | 28.7 | 18 | 52 | 0.84 (0.49–1.43) | 0.519 |
| DM | 89 | 45 | 50.6 | 11 | 12 | 0.85 (0.38–1.93) | 0.699 |
| RA | 55 | 7 | 12.7 | 2 | 16 | insufficient events |  |
| SSc | 55 | 15 | 27.3 | 0 | 3 | insufficient events |  |
| ASyS | 41 | 30 | 73.2 | 6 | 0 | insufficient events |  |
| SS | 39 | 35 | 89.7 | 2 | 1 | insufficient events |  |
| SLE | 30 | 15 | 50 | 9 | 8 | 1.23 (0.47–3.20) | 0.670 |
| UCTD | 27 | 10 | 37 | 5 | 3 | 3.06 (0.73–12.85) | 0.126 |
| CADM | 21 | 16 | 76.2 | 6 | 3 | 0.65 (0.16–2.62) | 0.548 |
| AAV | 16 | 0 | 0 | 0 | 12 | insufficient events |  |
| PM | 16 | 9 | 56.2 | 3 | 5 | 0.33 (0.08–1.42) | 0.138 |
| MCTD | 9 | 7 | 77.8 | 0 | 0 | insufficient events |  |
| SVV | 3 | 0 | 0 | 0 | 3 | insufficient events |  |
| AOSD | 1 | 0 | 0 | 0 | 1 | insufficient events |  |
| ALL ILD | 538 | 228 | 42.4 | 62 | 119 | 0.66 (0.48–0.90) | 0.008 |

Events (Ro52+)/(Ro52−), all-cause deaths among anti-Ro52-positive/negative patients. HR, hazard ratio; 95% CI, 95% confidence interval from Cox regression; P, Wald test P-value. 'insufficient events' = stratum with <5 deaths where HR was not estimable; ALL ILD is the pooled cohort of 538 patients. Small-vessel vasculitis (SVV) is an ANCA-associated vasculitis (AAV) subtype and is listed as a separate row for full transparency; AAV and SVV combined represent 19/538 (3.5%) patients.

Table S4. Baseline characteristics of 538 ILD patients stratified by Ro52 status, with standardized mean differences (SMD) and missing-data counts

| **Variable** | **Ro52 positive (N=228)** | **Ro52 negative (N=310)** | **SMD** | **P-value** | **N missing** |
| --- | --- | --- | --- | --- | --- |
| Age (years) | 56.7 ± 11.7 | 60.3 ± 14.5 | -0.27 | <0.001 | 0 |
| Female sex | 173/228 (75.9%) | 191/310 (61.6%) | 0.30 | <0.001 | 0 |
| BMI (kg/m2) | 21.9 ± 3.1 | 21.9 ± 3.3 | -0.01 | 0.941 | 0 |
| Ever smoker | 29/184 (15.8%) | 46/236 (19.5%) | -0.10 | 0.389 | 118 |
| CTD (vs Non-CTD) | 189/228 (82.9%) | 213/310 (68.7%) | 0.34 | <0.001 | 0 |
| FVC % predicted | 76.8 ± 19.0 | 78.9 ± 21.1 | -0.10 | 0.358 | 223 |
| FEV1 % predicted | 76.0 ± 19.7 | 80.1 ± 21.2 | -0.20 | 0.082 | 223 |
| TLC % predicted | 74.5 ± 13.5 | 74.4 ± 14.6 | 0.00 | 0.986 | 261 |
| DLCO % predicted | 51.0 ± 15.3 | 52.4 ± 18.4 | -0.09 | 0.466 | 265 |
| ANA positive | 177/223 (79.4%) | 117/292 (40.1%) | 0.87 | <0.001 | 23 |
| ANCA positive | 3/208 (1.4%) | 7/266 (2.6%) | -0.08 | 0.524 | 64 |
| Ro-60 positive | 89/223 (39.9%) | 26/291 (8.9%) | 0.74 | <0.001 | 24 |
| SSB positive | 18/222 (8.1%) | 2/290 (0.7%) | 0.38 | <0.001 | 26 |
| Creatine kinase (U/L) | 61 (36, 220) | 50 (31, 77) | 0.27 | <0.001 | 86 |
| Lactate dehydrogenase (U/L) | 290 (228, 390) | 246 (198, 339) | 0.28 | <0.001 | 78 |
| Alanine aminotransferase (U/L) | 24 (15, 45) | 19 (12, 33) | 0.26 | 0.001 | 77 |
| Aspartate aminotransferase (U/L) | 28 (20, 57) | 22 (18, 35) | 0.26 | <0.001 | 76 |
| C-reactive protein (mg/L) | 9 (4, 26) | 10 (5, 30) | -0.12 | 0.378 | 101 |
| IgG (g/L) | 14.3 (11.4, 17.9) | 12.7 (10.3, 16.1) | 0.37 | <0.001 | 114 |
| IgA (g/L) | 2.46 (1.83, 3.16) | 2.36 (1.70, 3.37) | 0.02 | 0.509 | 114 |
| IgM (g/L) | 1.29 (0.84, 1.89) | 1.08 (0.72, 1.61) | 0.25 | 0.007 | 115 |
| Complement C3 (g/L) | 0.95 (0.84, 1.07) | 1.01 (0.88, 1.15) | -0.30 | <0.001 | 115 |
| Complement C4 (g/L) | 0.18 (0.14, 0.22) | 0.20 (0.16, 0.25) | -0.33 | <0.001 | 115 |
| Malignancy | 17/228 (7.5%) | 30/310 (9.7%) | -0.08 | 0.455 | 0 |

SMD, standardized mean difference (continuous: mean diff / pooled SD; binary: proportion diff / pooled SD). P from Welch's t-test or Wilcoxon rank-sum test (continuous) or χ²/Fisher (categorical). Missing counts are total N with absent values in each variable. ANA, anti-nuclear antibody; ANCA, anti-neutrophil cytoplasmic antibody; Ro-60, anti-Ro-60; SSB, anti-SS-B; CK, creatine kinase; LDH, lactate dehydrogenase; ALT, alanine aminotransferase; AST, aspartate aminotransferase; CRP, C-reactive protein; IgG/A/M, immunoglobulin; C3/C4, complement component 3/4; CTD, connective tissue disease; FVC, forced vital capacity; FEV1, forced expiratory volume in 1 s; TLC, total lung capacity; DLCO, diffusing capacity for carbon monoxide.

Table S5. Critical care and glucocorticoid exposure by Ro52 status (n = 538)

| **Variable** | **Level** | **Ro52+** | **Ro52−** | **P-value** |
| --- | --- | --- | --- | --- |
| ICU admission | Yes | 41/228 (18.0%) | 74/310 (23.9%) | 0.124 |
| ICU admission | No | 187/228 (82.0%) | 236/310 (76.1%) |  |
| Endotracheal intubation | Yes | 39/228 (17.1%) | 67/310 (21.6%) | 0.234 |
| Endotracheal intubation | No | 189/228 (82.9%) | 243/310 (78.4%) |  |
| Mechanical ventilation | Yes | 42/228 (18.4%) | 73/310 (23.5%) | 0.184 |
| Mechanical ventilation | No | 186/228 (81.6%) | 237/310 (76.5%) |  |
| ECMO | Yes | 2/202 (1.0%) | 2/262 (0.8%) | 1.000 |
| ECMO | No | 200/202 (99.0%) | 260/262 (99.2%) |  |
| Methylprednisolone dose category | 0 | 62/186 (33.3%) | 99/232 (42.7%) | 0.140 |
| Methylprednisolone dose category | 1 | 56/186 (30.1%) | 63/232 (27.2%) |  |
| Methylprednisolone dose category | 2 | 68/186 (36.6%) | 70/232 (30.2%) |  |

Percentages use the non-missing denominator for each variable (shown as n/N). ICU admission, intubation, mechanical ventilation and ECMO are severe-illness indicators/treatments and were not entered as covariates in the main Cox models. Methylprednisolone dose categories 0/1/2 correspond to increasing cumulative glucocorticoid exposure (median daily equivalent ≈14/40/77 mg). ECMO had 74 missing values. ICU, intensive care unit; ECMO, extracorporeal membrane oxygenation.

Table S6. All-cause survival by underlying disease (n = 538)

| **Disease group** | **N** | **Deaths** | **Death rate (%)** | **Median OS (d) [95% CI]** | **1-yr survival (%)** | **HR (95% CI) vs Non-CTD** | **P-value** |
| --- | --- | --- | --- | --- | --- | --- | --- |
| IIM | 167 | 46 | 27.5 | NR | 76.6 | 0.45 (0.31–0.66) | <0.001 |
| Non-CTD | 136 | 70 | 51.5 | 1264 (428–NR) | 60.3 | 1.00 (ref) | — |
| RA | 55 | 18 | 32.7 | NR | 83.6 | 0.51 (0.30–0.86) | 0.011 |
| SSc | 55 | 3 | 5.5 | NR | 100 | 0.08 (0.02–0.24) | <0.001 |
| SS | 39 | 3 | 7.7 | NR | 97.4 | 0.11 (0.03–0.34) | <0.001 |
| SLE | 30 | 17 | 56.7 | 78 (18–NR) | 46.7 | 1.27 (0.74–2.15) | 0.384 |
| UCTD | 27 | 8 | 29.6 | NR | 85.2 | 0.47 (0.23–0.97) | 0.042 |
| AAV | 16 | 12 | 75 | 1051 (27–NR) | 68.8 | 1.48 (0.80–2.73) | 0.212 |
| MCTD | 9 | 0 | 0 | NR | 100 | NR (0 events) | 0.993 |
| SVV | 3 | 3 | 100 | 12 (11–NR) | 0 | NR (n too small) | — |
| AOSD | 1 | 1 | 100 | 18 (NA–NR) | 0 | NR (n too small) | — |

Deaths, number of all-cause deaths; Death rate (%), deaths/N; Median OS (d), median overall survival in days [95% CI]; 1-yr survival (%), Kaplan-Meier 1-year survival; HR (95% CI) vs Non-CTD, hazard ratio vs non-CTD-ILD reference; P, log-rank P-value. NR, not reached; NA, not applicable. Small-vessel vasculitis (SVV) is an AAV subtype and is listed separately; AAV and SVV combined represent 19/538 (3.5%) patients.

**Table S7A. Four anti-MDA5/anti-Ro52 combinations in the myositis-antibody-tested subset (n = 372 with evaluable anti-Ro52 results, from 375 tested).**

| **Combination** | **N** | **Deaths** | **Mortality (%)** | **HR (95% CI)** | **P-value** |
| --- | --- | --- | --- | --- | --- |
| MDA5−/Ro52− (reference) | 133 | 11 | 8.3 | 1.00 (reference) | — |
| MDA5−/Ro52+ | 164 | 5 | 3.0 | 0.43 (0.15–1.27) | 0.126 |
| MDA5+/Ro52− | 40 | 7 | 17.5 | 2.61 (0.99–6.88) | 0.052 |
| MDA5+/Ro52+ | 35 | 10 | 28.6 | 4.52 (1.88–10.88) | <0.001 |

**Table S7B. Full interaction-model output for anti-MDA5 × anti-Ro52 in the myositis-antibody-tested subset (n = 372; 33 events in all models).**

| **Model term** | **β** | **SE** | **HR (95% CI)** | **P-value** |
| --- | --- | --- | --- | --- |
| Ro52+ (main effect) | −0.84 | 0.55 | 0.43 (0.15–1.27) | 0.126 |
| MDA5+ (main effect) | 0.96 | 0.49 | 2.61 (0.99–6.88) | 0.052 |
| Ro52+ × MDA5+ (interaction) | 1.39 | 0.74 | 3.99 (0.94–16.94) | 0.060 |
| Ro52+ (main effect), adjusted for age and sex | −0.93 | 0.55 | 0.39 (0.13–1.15) | 0.089 |
| MDA5+ (main effect), adjusted for age and sex | 1.05 | 0.50 | 2.86 (1.07–7.65) | 0.036 |
| Ro52+ × MDA5+ (interaction), adjusted for age and sex | 1.57 | 0.74 | 4.81 (1.13–20.42) | 0.033 |
| Likelihood-ratio test for Ro52 × MDA5 (age/sex-adjusted), n = 372, 33 events | — | — | — | 0.030 |

HRs are from Cox proportional-hazards models, with MDA5−/Ro52− as the reference category (Table S7A, four-combination model) or from the unadjusted interaction model (Table S7B, survival ~ Ro52 × MDA5). The myositis-antibody-tested subset comprised 375 patients with 34 deaths. Three anti-MDA5-positive patients had unscorable anti-Ro52 assay results despite attempted testing and were excluded from the four-combination and unadjusted interaction models; one of these three patients died, so these models included 372 patients and 33 deaths. Two patients had implausible registry age values (0.38 and 0.51 years); review of their records (age at disease onset and interval to diagnosis) identified decimal-shift entry errors, and their ages were corrected to 38 and 51 years, so all models used the same 372 patients and 33 deaths. Likelihood-ratio test for the Ro52 × MDA5 interaction: P = 0.055 (unadjusted) and P = 0.030 (age/sex-adjusted). The unadjusted models are presented because the myositis-antibody-tested subset has limited covariate coverage and contributed only 33 analyzable events.

**Table S8. Baseline characteristics of patients lost to follow-up versus those with known survival status.**

| **Variable** | **Lost to follow-up (n=131)** | **Known status (n=407)** | **P-value** |
| --- | --- | --- | --- |
| Age (years) | 60.2 ± 12.3 | 58.3 ± 13.8 | 0.178 |
| Female sex | 96/131 (73.3%) | 268/407 (65.8%) | 0.140 |
| BMI (kg/m²) | 21.5 ± 3.1 | 22.0 ± 3.2 | 0.064 |
| Ever smoker | 20/116 (17.2%) | 55/304 (18.1%) | 0.951 |
| CTD (vs Non-CTD) | 110/131 (84.0%) | 292/407 (71.7%) | 0.007 |
| Ro52 positive | 54/131 (41.2%) | 174/407 (42.8%) | 0.836 |
| FVC % predicted | 78.0 ± 21.2 | 77.9 ± 19.9 | 0.924 |
| DLCO % predicted | 52.7 ± 14.6 | 51.4 ± 18.0 | 0.498 |
| Malignancy | 7/131 (5.3%) | 40/407 (9.8%) | 0.161 |

Ro52 positivity was similar between groups (41.2% vs 42.8%, P = 0.836), supporting non-differential censoring with respect to Ro52 status. P-values from Welch’s t-test, chi-square, or Fisher’s exact test as appropriate.

**Table S9. Sensitivity analyses addressing ICU-related selection: association of anti-Ro52 with all-cause mortality (m = 20 imputation)**

| **Analysis** | **N** | **HR (95% CI)** | **P-value** |
| --- | --- | --- | --- |
| Model 6 (full cohort) | 538 | 0.72 (0.52–0.99) | 0.045 |
| Model 6 + ICU adjustment | 538 | 0.91 (0.65–1.28) | 0.594 |
| Model 6 excluding ICU admissions | 423 | 0.59 (0.35–1.01) | 0.057 |

Abbreviations: CI, confidence interval; HR, hazard ratio; ICU, intensive care unit; MI, multiple imputation. Model 6 included age, sex, BMI, smoking, malignancy, FVC%, and DLCO%, with MI (m = 20). ICU status was recorded for all patients (n = 538; 115 ICU admissions). The full cohort was analyzed with and without adjustment for ICU status, and separately after exclusion of ICU admissions (n = 423).
